# Allele-Specific Knockout by CRISPR/Cas to Treat Autosomal Dominant Retinitis Pigmentosa Caused by the G56R Mutation in NR2E3

**DOI:** 10.3390/ijms22052607

**Published:** 2021-03-05

**Authors:** Michalitsa Diakatou, Gregor Dubois, Nejla Erkilic, Carla Sanjurjo-Soriano, Isabelle Meunier, Vasiliki Kalatzis

**Affiliations:** 1 INM, University of Montpellier, Inserm, 34091 Montpellier, France; michalitsa.diakatou@inserm.fr (M.D.); gregor.dubois@inserm.fr (G.D.); nejla.erkilic@inserm.fr (N.E.); carla.sanjurjo-soriano@inserm.fr (C.S.-S.); isabelannemeunier@yahoo.fr (I.M.); 2 National Reference Centre for Inherited Sensory Diseases, University of Montpellier, CHU, 34295 Montpellier, France

**Keywords:** inherited retinal dystrophies, autosomal dominant retinitis pigmentosa, rod-cone dystrophy, NR2E3, CRISPR/Cas, photoreceptor, allele-specific, knockout, iPSC, retinal organoids

## Abstract

Retinitis pigmentosa (RP) is an inherited retinal dystrophy that causes progressive vision loss. The G56R mutation in NR2E3 is the second most common mutation causing autosomal dominant (ad) RP, a transcription factor that is essential for photoreceptor development and maintenance. The G56R variant is exclusively responsible for all cases of *NR2E3*-associated adRP. Currently, there is no treatment for *NR2E3*-related or, other, adRP, but genome editing holds promise. A pertinent approach would be to specifically knockout the dominant mutant allele, so that the wild type allele can perform unhindered. In this study, we developed a CRISPR/Cas strategy to specifically knockout the mutant G56R allele of *NR2E3* and performed a proof-of-concept study in induced pluripotent stem cells (iPSCs) of an adRP patient. We demonstrate allele-specific knockout of the mutant G56R allele in the absence of off-target events. Furthermore, we validated this knockout strategy in an exogenous overexpression system. Accordingly, the mutant G56R-CRISPR protein was truncated and mis-localized to the cytosol in contrast to the (peri)nuclear localizations of wild type or G56R NR2E3 proteins. Finally, we show, for the first time, that G56R iPSCs, as well as G56R-CRISPR iPSCs, can differentiate into NR2E3-expressing retinal organoids. Overall, we demonstrate that G56R allele-specific knockout by CRISPR/Cas could be a clinically relevant approach to treat *NR2E3*-associated adRP.

## 1. Introduction

Inherited retinal dystrophies (IRDs) are a group of disorders characterized by progressive vision loss. This is due to degeneration of the light-sensing cells of the retina, the photoreceptors. Rod-cone dystrophy is the most common IRD form, more commonly known as Retinitis Pigmentosa (RP), which affects ~1/4000 people worldwide [1]. RP is initially characterized by night blindness and peripheral visual field loss, and subsequently by central vision loss, due to the sequential degeneration of rod and cone photoreceptors [2]. RP is caused by mutations in over 80 genes and it can be transmitted in autosomal dominant (adRP; one mutant allele is sufficient for disease manifestation), autosomal recessive (two mutant alleles are required), and X-linked inheritance patterns [1]. Furthermore, mutations in the same IRD gene can cause both autosomal dominant and recessive forms, and mutation hotspots can result in elevated disease prevalence in certain populations [3]. The most common mutation that is responsible for adRP is the missense mutation c.68C>A (p.Pro23His; P23H) in *RHO*, the gene encoding rhodopsin, the visual pigment of rod photoreceptors. The second most common mutation for adRP is the c.166G>A (p.Gly56Arg; G56R) mutation in *NR2E3*. The G56R mutation is exclusively responsible for all *NR2E3*-associated adRP cases and, alone, accounts for 1–2% of total adRP cases in America and 3.5% in Europe [4,5,6].

NR2E3 (Nuclear Receptor subfamily 2 group E member 3) is a photoreceptor-specific transcription factor and key player in the development and maintenance of rod photoreceptors [7,8,9]. More specifically, NR2E3 acts in a complex with CRX, NRL, and NR1D1 to promote the transcription of rod genes, such as *RHO*, while repressing the transcription of cone genes [8,10]. NR2E3 has a typical nuclear receptor structure with two main domains, a DNA Binding Domain (DBD) and a Ligand Binding Domain (LBD) [11]. In addition to DNA binding, the DBD is responsible for the interaction of NR2E3 with CRX [9]. By contrast, the LBD mediates the formation of NR2E3 homodimers, which are necessary in transcriptional repression [12,13]. The highly conserved glycine residue at position 56 is localized within the DBD of NR2E3. The disease mechanism of the G56R variant remains elusive. Some of the hypotheses are that the G56R mutation reduces DNA binding and subsequent *RHO* activation [14,15,16], weakens CRX binding [16], or decreases homodimerization [15] of NR2E3.

Currently, there is no treatment for *NR2E3*-associated adRP or other adRPs. However, the advent of the powerful CRISPR/Cas technology provides hope for genome editing as a therapeutic approach [17,18,19]. The CRISPR/Cas system comprises two elements, a Cas endonuclease and a 20-nt guide RNA (gRNA). The gRNA is situated next to a 3-nt sequence, which is known as a protospacer adjacent motif (PAM) [17]. The most commonly used Cas is Cas9 from *Streptococcus pyogenes* (Sp), which recognizes an NGG PAM sequence. The combination of the PAM and gRNA molecule guides the Cas9 to the target sequence in the host DNA, where it induces a double-strand break (DSB). The DSB is then repaired by the cell machinery by one of two main pathways [20]. The first pathway is homologous-directed repair (HDR), which takes place during the S/G2 phase of dividing cells [21]. HDR is exploited for gene correction by providing a DNA sequence repair template along with the CRISPR/Cas system [22]. The second pathway is non-homologous end joining (NHEJ), which is recruited during all phases of the cell cycle in the absence of a repair template.

In most cases where genome-editing is envisaged, the ideal choice would be to correct a mutant allele by the HDR pathway. However, this can be challenging in post-mitotic cells, such as photoreceptors. By contrast, knocking out a mutant allele could be efficient and clinically relevant, especially for an autosomal dominant disorder. This approach takes advantage of the susceptibility to error of the NHEJ pathway, namely the introduction of small insertions or deletions (indels) at the cleavage site. These indels may lead to a frameshift and a premature termination codon (PTC) [23]. The mRNA may be subjected to nonsense-mediated decay (NMD), depending on where the premature stop codon emerges. A risk of mutant allele ablation is haploinsufficiency, i.e., the insufficiency of a single copy of the target gene to produce adequate protein levels for cell function. However, in the case of *NR2E3*, this is not likely an issue. Homozygous mutations in *NR2E3* give rise to an autosomal recessive IRD called Enhanced S Cone Syndrome (ESCS) and one of the most common is a loss-of-function splice site mutation in intron 1 that only spares the first exon [24]. As is common for autosomal recessive IRDs, heterozygous carriers of *NR2E3* mutations are healthy subjects, as are heterozygous littermates of the *Nr2e3^−/−^* mouse models [25,26]. Taken together, these data suggest that one wild type (WT) *NR2E3* allele is sufficient for the correct development and function of rod photoreceptors.

Therefore, we aimed to specifically knockout the G56R allele as a potential treatment for *NR2E3*-associated adRP. To this end, we designed a CRISPR/Cas genome-editing strategy to specifically ablate the mutant allele in induced pluripotent stem cells (iPSCs) of an *NR2E3*-associated adRP patient. Here, we report on the success of this approach, and on the pluripotency and retinal differentiation potential of the genome-edited iPSCs.

## 2. Results

### 2.1. Reprogramming G56R Fibroblasts to iPSCs

**Figure 1:**
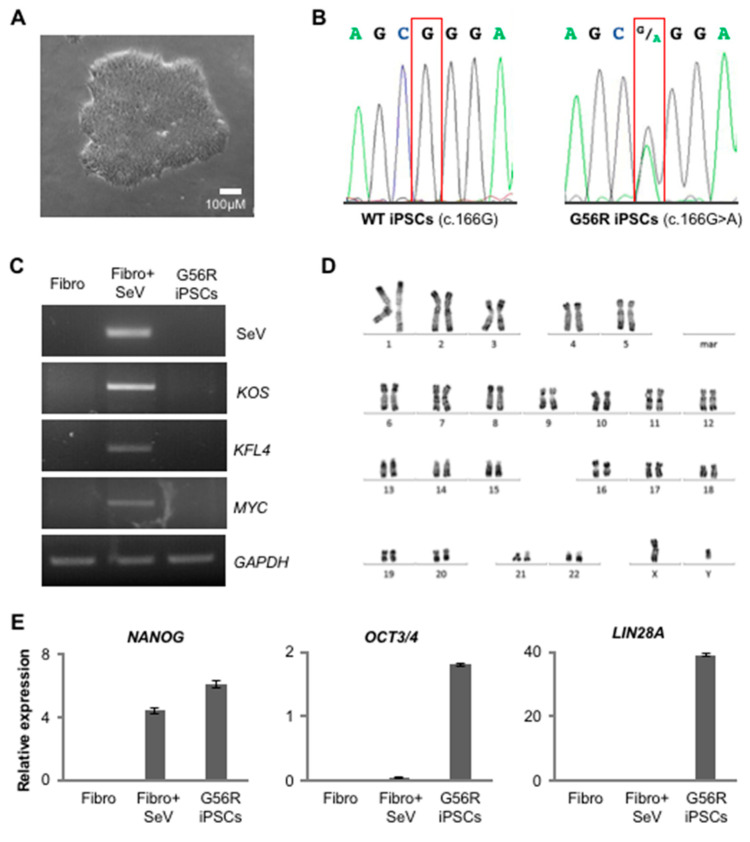

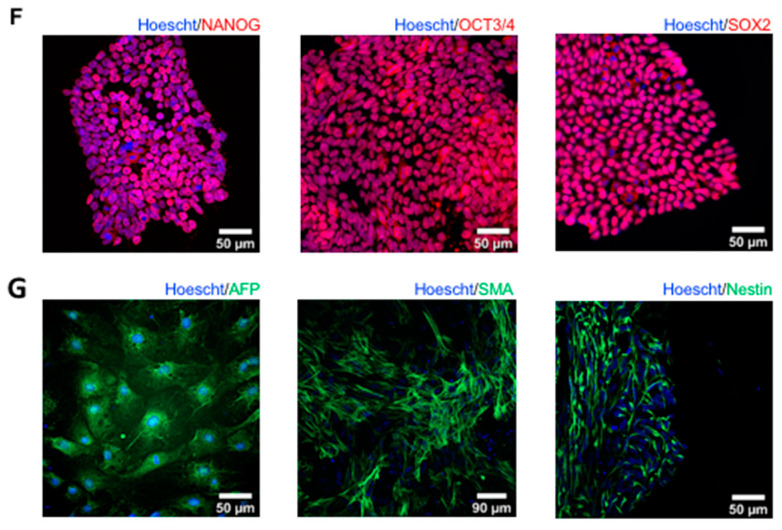
Characterization of the G56R induced pluripotent stem cells (iPSC) line from the *NR2E3*-adRP patient. (**A**) Bright-field microscopy of a G56R iPSC colonies. (**B**) Sanger sequencing of exon 2 of *NR2E3* showing a G at cDNA position 166 in the DNA of wild type (WT) iPSC and the G>A transition at c.166 in the G56R iPSC line. (**C**) RT-PCR detection of the reprogramming vectors in RNA from non-transduced patient fibroblasts (Fibro; negative control), fibroblasts transduced with the Sendai virus vectors (Fibro + SeV; positive control) and G56R iPSCs using primers specific for either the vector backbone (SeV) or for each reprogramming cassette: polycistronic *KLF4*, *OCT3/4,* and *SOX2* (KOS), or monocistronic *KLF4* and *MYC*. Primers for the housekeeping gene *GAPDH* were used as a positive control for the PCR reaction. (**D**) Karyotype analysis of the G56R iPSC line showing normal chromosomal number and structure. (**E**) qPCR analysis of the expression of the host pluripotency genes *NANOG* (left), *OCT3/4* (middle), and *LIN28A* (right) in cDNA from non-transduced patient fibroblasts (Fibro; negative control), fibroblasts transduced with the Sendai virus vectors (Fibro + SeV) and G56R iPSCs. The results are expressed as mean ± SEM (*n* = 3). (**F**) Immunofluorescence (IF) studies of the expression of endogenous NANOG (left), OCT3/4 (middle) and SOX2 (right) in G56R iPSCs; nuclei labelled in blue. (**G**) IF studies following embryoid body differentiation of G56R iPSCs for the expression of the germ layer markers Alpha Fetal Protein (AFP) (endoderm; left), Smooth Muscle Actin (SMA) (mesoderm; middle), and Nestin (ectoderm; right).

We isolated dermal fibroblasts from a skin biopsy of an *NR2E3*-associated adRP patient in order to develop a CRISPR/Cas-mediated knockout approach for the G56R mutation. We reprogrammed the fibroblasts using non-integrative Sendai virus (SeV) vectors carrying Yamanaka’s transcription factor cocktail *KLF4*, *OCT3/4*, *SOX2*, and *c-MYC* [27]. Emerging iPSCs showed a typical morphology that consists of tightly packed cells with a distinct border (Figure 1A). Sanger sequencing confirmed the presence of the c.166G>A mutation in the G56R iPSCs as compared to WT iPSCs (Figure 1B). At passage (P) 12, RT-PCR analysis of the exogenous pluripotency genes determined the clearance of the SeV vectors in comparison to non-transduced (negative control) and SeV-transduced (positive control) fibroblasts (Figure 1C). We observed a normal karyotype of the G56R iPSCs at P11, being indicative of genetic stability (Figure 1D). By qPCR analysis, we detected the expression of the host pluripotency genes *NANOG*, *OCT 3/4*, and *LIN28A* in comparison to the lack of expression in non-transduced and SeV-transduced G56R fibroblasts (Figure 1E); NANOG expression was already detectable in SeV-transduced fibroblasts, consistent with our previous observations [28,29]. Pluripotency was further confirmed by immunofluorescence (IF) staining for NANOG, OCT3/4, and SOX2 (Figure 1F). In addition, we validated the ability of the G56R iPSC to differentiate into the three germ layers using an embryoid body (EB) differentiation assay and IF staining to detect specific markers: Alpha Fetal Protein (AFP) for endoderm, Smooth Muscle Actin (SMA) for mesoderm, and Nestin for ectoderm (Figure 1G). Taken together, the generated G56R iPSC line passed all of the quality controls assessing pluripotency and integrity.

In conclusion, we generated a pluripotent and genetically stable G56R iPSC line from an *NR2E3*-associated adRP patient, which could be used for therapeutic development.

### 2.2. Designing a G56R-Specific Knockout Strategy

**Figure 2:**
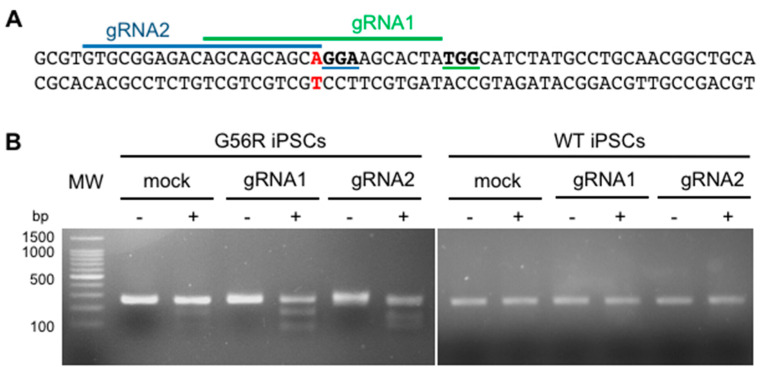
Design and efficiency testing of gRNAs. (**A**) Sequence of exon 2 of *NR2E3* in the DNA of the *NR2E3*-adRP patient showing the position of the c.166G>A mutational site in red. The position of gRNA1 is indicated by a green line and the corresponding NGG PAM sequence is in bold and underlined in green. The position of gRNA2 is indicated by a blue line and the corresponding NGA PAM sequence is indicated in bold and underlined in blue. (**B**) Gel electrophoresis of the T7E1 assay in G56R and WT iPSCs that were mock-transfected or transfected with gRNA1 or gRNA2. The minus sign indicates the absence, and the plus sign indicates the presence, of T7E1. Molecular weight (MW) marker, 100 bp ladder.

The challenge of a G56R-specific knockout strategy was to design allele-specific gRNAs that would not target the wild type (WT) allele, which could only be achieved by gRNAs that spanned the mutational site. We used three online software for gRNA design: CCTOP (https://crispr.cos.uni-heidelberg.de), CRISPOR (http://crispor.tefor.net), and MIT CRISPR online design tool (https://zlab.bio/guide-design-resources, no longer available), and identified two overlapping gRNAs that fit our criteria (Figure 2A). The first, gRNA1, had an NGG PAM sequence, which could be recognized by the SpCas9 as well as by its enhanced specificity variant, eSpCas9, which has reduced off-target effects [30]. The second gRNA, gRNA2, had an NGA PAM sequence, which could be recognized by the engineered VQR variant of SpCas9, which was created to increase the repertoire of genomic target sequences [31]. When designing gRNA2, we did not add a mismatched G in its 5′, which has been described to enhance transcription that was driven by the U6 promoter in the expression plasmid [32], because the first nucleotide was already a G. Similarly, we did not include an extra G for gRNA1 that pairs with the eSpCas9, because we previously showed that this reduces its activity [33].

We next assessed the ability of the two gRNAs to selectively target the G56R allele. We cloned each gRNA into the corresponding Cas9 plasmid, which contained an enhanced green fluorescent protein (EGFP) tag, and nucleofected WT and G56R iPSCs. Two-days post-transfection, we isolated genomic DNA and performed a T7 endonuclease 1 (T7E1) assay. T7E1 digests DNA when there is mismatch between the two strands, as can occur if there is a frameshift mutation in one of the alleles. A positive digestion result is indicated by multiple bands after gel electrophoresis. The T7E1 assay was positive for both gRNAs in the patient iPSCs, indicating that at least one of the alleles had been targeted, as shown in (Figure 2B). Two faint bands were also present in the non-transfected patient cells, likely corresponding to the mismatch due to the heterozygous c.166G>A mutation. By contrast, the T7E1 assay was negative for both gRNAs in the WT iPSCs, which indicated that the WT allele was not targeted.

Overall, we identified two gRNAs that specifically targeted the mutant G56R allele. For all subsequent experiments, we retained gRNA1 due to the higher specificity and lower off-target effects of eSpCas9.

### 2.3. Knocking Out the G56R Allele in Patient iPSCs

**Figure 3:**
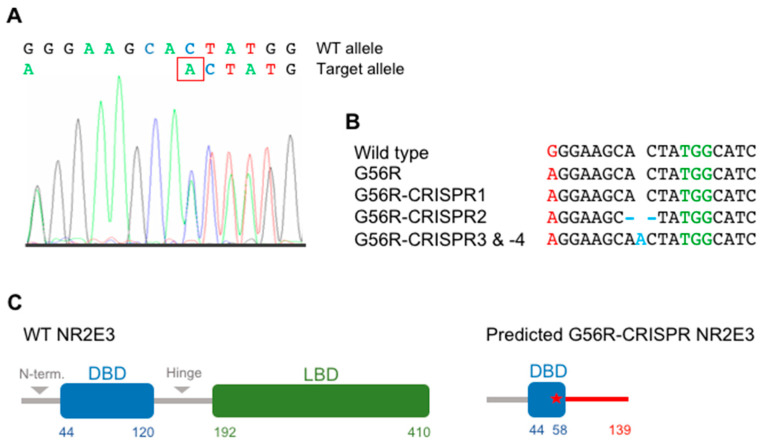
Generation of G56R-CRISPR iPSC lines. (**A**) Representative electropherogram of the sequence flanking the DSB site in the DNA of the G56R-CRISPR3 and -4 iPSC clones. The sequence begins at the c.166G>A mutational site. The introduced nucleotide (c.174insA) is boxed in red. (**B**) Alignment of the Sanger sequencing results for the same region shown in (**A**) in the wild type, G56R and four G56R-CRISPR IPSC clones. The mutational site is shown in red. The indels are indicated in blue. The PAM sequence is shown in green. (**C**) Graphical representation of the protein structure of 410 aa WT NR2E3 (left) with the DNA binding domain (DBD) shown in blue, the ligand binding domain (LBD) shown in green and the N-terminus and the hinge region are shown in grey. The predicted effect on NR2E3 (right) following introduction of the frameshift mutation on the G56R (indicated by a red star in the truncated DBD) mutant allele. The red bar indicates a non-NR2E3 protein sequence prior to the premature termination at 139 aa.

We transfected G56R iPSCs with the gRNA1-expressing Cas9 plasmid to generate a clonal genome-edited G56R iPSC line. The EGFP-positive cells were single cell-sorted by FACS 48-h post-transfection. Two weeks post-sorting, four surviving iPSC colonies emerged. We expanded these clones and Sanger sequenced the mutational site in exon 2 of *NR2E3* (Figure 3A). One clone (G56R-CRISPR1) showed no change following genome-editing, whereas the other three clones (G56R-CRISPR2, -3 and -4) showed the introduction of indels in the mutant allele (Figure 3B). The mutations were introduced 3-bp upstream of the PAM sequence, at the exact spot where Cas9 is predicted to induce a DSB. The clone G56R-CRISPR2 carried a deletion of two nucleotides (c.173_174delAC), whereas the clones G56R-CRISPR3 and -4 had the same insertion of one nucleotide (c.174insA). Thus, we only retained the clones G56R-CRISPR2 and -3 for further experiments. We verified the sequencing results by individually sub-cloning each allele of the CRISPR clones into the pGEM-T Easy vector system and then re-sequencing. We sequenced at least 10 colonies for each clone and identified a 1:1 detection ratio of G56R to WT alleles (Supplementary Figure S1).

A predictive analysis of the protein sequence coded by the G56R-CRISPR2 and -3 clones identified a frameshift that resulted in a PTC, p.(His58Leufs*82) for G56R-CRISPR2 and p.(His58Glnfs*83) for G56R-CRISPR3. It is noteworthy that the new open reading frame was predicted to be identical for both G56R-CRISPR2 and -3, with the exception that the latter contained one extra amino acid (aa) residue at the point of insertion. According to the predicted protein sequences of the G56R-CRISPR2 and -3 clones, over half of the DBD (residues 44–120; www.uniprot.org) was removed, as well as the entire LBD (residues 192–410; [12]) (Figure 3C).

Taken together, the genome-edited G56R-CRISPR iPSC clones carried allele-specific indels, which were predicted to disrupt the formation of a full-length protein.

### 2.4. Characterizing the Pluripotency and Stability of G56R-CRISPR iPSC Lines

**Figure 4:**
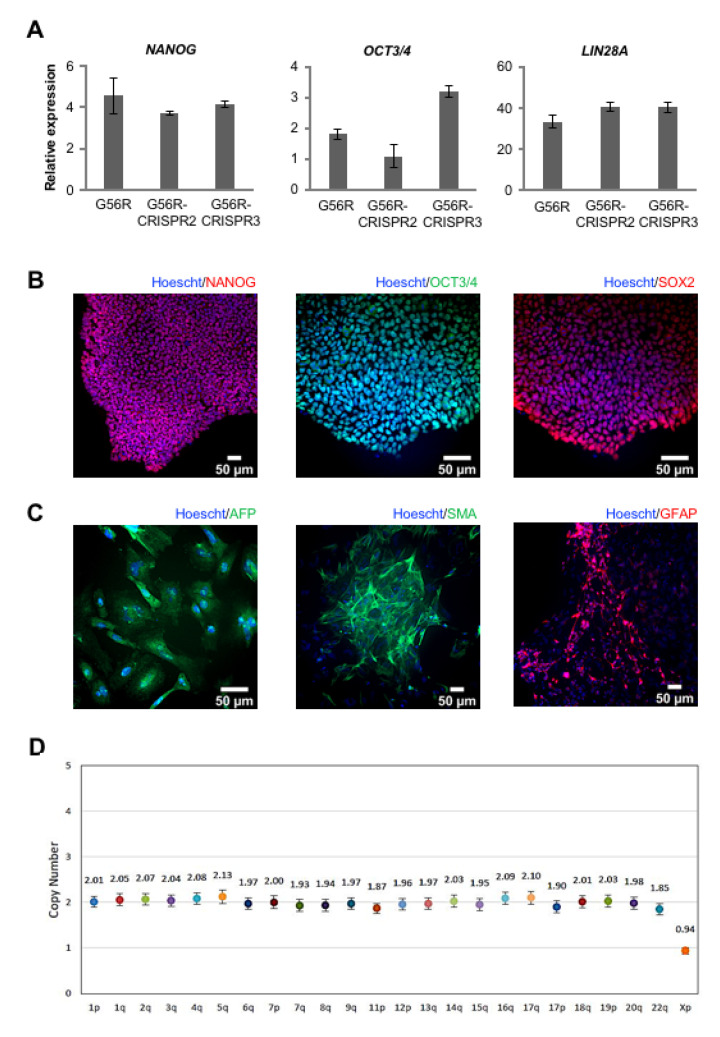
Characterization of the G56R-CRISPR2 and -3 iPSC lines. (**A**) qPCR analysis of the expression of the host pluripotency genes *NANOG* (left), *OCT3/4* (middle), and *LIN28A* (right) in cDNA from G56R and G56R-CRISPR2 and -3 iPSCs. Results are expressed as mean ± SEM (*n* = 3). (**B**) Representative images of IF analysis of the expression of endogenous NANOG (left; G56R-CRISPR3), OCT3/4 (middle; G56R-CRISPR2), and SOX2 (right; G56R-CRISPR2) in G56R-CRISPR2 and -3 iPSCs; nuclei labelled in blue. (**C**) Representative images of IF analysis following embryoid body differentiation of G56R-CRISPR2 and -3 iPSCs for the expression of the germ layer markers AFP (endoderm; left; G56R-CRISPR3), SMA (mesoderm; middle; G56R-CRISPR3), and glial fibrillary acidic protein (GFAP) (ectoderm; right; G56R-CRISPR2). (**D**) Representative digital qPCR analysis of the G56R-CRISPR3 iPSC line for the presence of the most commonly rearranged chromosomal regions detected in iPSCs. A copy number of ~2 for the autosomes, and 1 for the sex chromosomes, is indicative of stability.

Afterwards, we assayed whether the G56R-CRISPR2 and -3 lines retained their iPSC characteristics and genetic stability, in comparison to the parental G56R iPSC line. By qPCR analysis, we detected the expression of the pluripotency genes *NANOG*, *OCT3/4,* and *LIN28A* in the G56R-CRISPR2 and -3 lines at similar levels to the parental G56R line (Figure 4A) and, by IF analysis, the expression of the pluripotency markers NANOG, OCT3/4, and SOX2 (Figure 4B). In addition, the G56R-CRISPR2 and -3 lines differentiated into the three germ layers, as determined by IF analysis of AFP expression for endoderm, SMA for mesoderm, and glial fibrillary acidic protein (GFAP) for ectoderm (Figure 4C). Moreover, we tested genetic stability of the G56R-CRISPR2 and -3 iPSC lines by a digital PCR test of the copy number variant (CNV) of the most commonly rearranged chromosomal regions reported in iPSCs [34]. A CNV of 2 was detected for the autosomes, and a CNV of 1 for the X chromosome, in both lines indicating that the G56R-CRISPR2 and -3 iPSCs were genetically stable (Figure 4D).

Finally, we evaluated potential off-target events that may have been introduced in the G56R-CRISPR clones. We PCR-amplified the off-target sites for gRNA1 predicted by the CRISPOR software from the DNA of the G56R-CRISPR2 and -3 lines while using specific primers and analyzed the amplicons by Sanger sequencing; to have a complete overview of potential off-target events, we additionally tested G56R-CRISPR4. The sequences for each of these sites in the G56R-CRISPR2, -3 and -4 clones were identical to that of WT, demonstrating the absence of off-target events, as shown in Supplementary Figure S2.

In conclusion, following eSpCas9-mediated genome-editing, the resulting G56R-CRISPR iPSC lines retained their pluripotency and genetic stability, and they did not harbor off-target events.

### 2.5. Validating Allele-Specific Knockout of Mutant G56R NR2E3 at the Protein Level

We tested *NR2E3* expression levels in iPSCs by qPCR to validate our allele-specific knockout strategy at the mRNA level. However, this was inconclusive, as, firstly, *NR2E3* was expressed at very low levels (>30 cycles) and, secondly, we were not able to differentiate between the expression of the WT, G56R, and G56R-CRISPR2 and -3 alleles. Accordingly, we moved on to directly evaluate the effect of our genome-editing strategy at the protein level while using a cell-based overexpression assay. We first amplified WT *NR2E3* cDNA from a commercial pool of retinal cDNA by PCR and then cloned the amplicon into the pGEM-T Easy vector system. Using this construct as a base, we performed site-directed mutagenesis and generated two additional plasmids, one containing the G56R mutation and the other, the G56R-CRISPR2 frameshift mutation (we did not introduce the G56R-CRISPR3 and -4 frameshift mutation, as the predicted protein truncation was identical to G56R-CRISPR2). Subsequently, we sub-cloned the three *NR2E3* variants (WT, G56R and G56R-CRISPR2) into the pcDNA3 expression plasmid. The constructs were then separately transfected into HEK293 and COS7 cell lines and we performed western blot and IF studies 48 h post-transfection.

**Figure 5:**
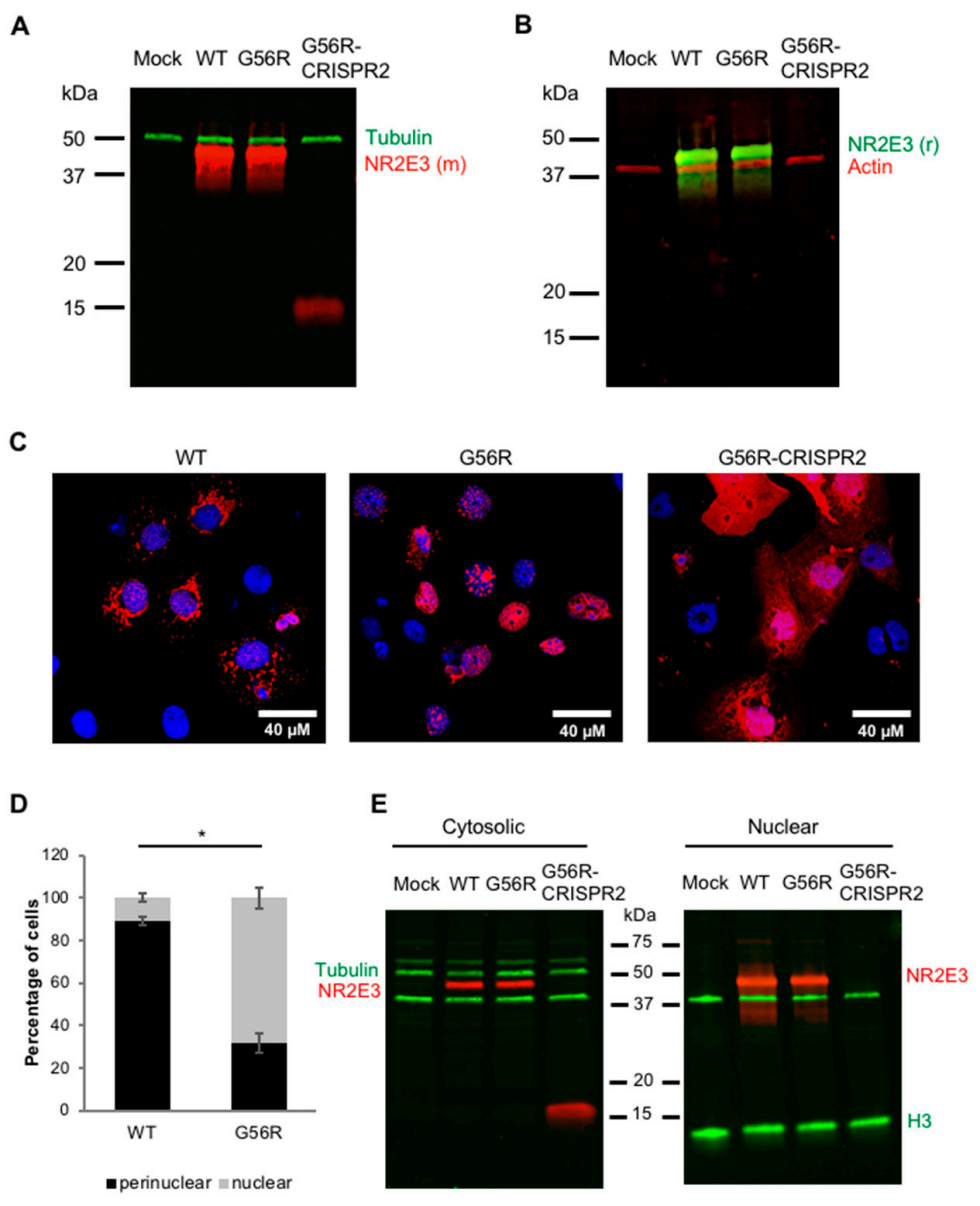
Effect of mutant allele knockout on NR2E3 expression. Western blot analysis of HEK293 cells mock-transfected or transfected with the pCDNA3 constructs expressing WT, G56R or G56R-CRISPR2 NR2E3 and hybridized with (**A**) a mouse monoclonal anti-NR2E3 antibody (NR2E3 (m); in red) or (**B**) a rabbit polyclonal NR2E3 antibody (NR2E3 (r); in green). Both anti-NR2E3 antibodies detected a 45-kDa protein in WT and G56R-expressing cells. The mouse monoclonal anti-NR2E3 antibody detected a truncated 16-kDa protein in the G56R-CRISPR2-expressing cells (**A**). Loading controls, an anti-tubulin antibody detected a 55-kDa protein (in green) (**A**); an anti-actin antibody detected a 42-kDa protein (in red) (**B**). (**C**) Representative images following IF analysis of COS7 cells transfected with the pcDNA3 constructs expressing wild type (left), G56R (middle) or G56R-CRISPR2 (right) NR2E3 in red; nuclei labelled in blue. (**D**) Quantification of the percentage of COS7 cells transfected with the pCDNA3 constructs expressing wild type or G56R NR2E3 showing the mixed perinuclear and nuclear versus mainly nuclear localization profiles. Results are expressed as mean ± SEM of three independent experiments (*n* = 3; * *p* < 0.05; Mann and Whitney test); each experimental value represented the mean of five separate fields. (**E**) Western blot analysis following differential centrifugation of cytosolic (left) and nuclear (right) fractions of HEK293 cells mock-transfected (lanes 1) or transfected with the pCDNA3 constructs expressing WT (lanes 2), G56R (lanes 3) or G56R-CRISPR2 (lanes 4) NR2E3. Bands corresponding to 45-kDa NR2E3 were detected in both fractions for WT and G56R-transfected cells, whereas a 16-kDa protein was only detected in the cytosolic fraction of G56R-CRISPR2-transfected cells. To control for fraction purity, both of the membranes were hybridized with anti-tubulin (55 kDa; in green) and anti-histone H3 (15 kDa; in green). A non-specific 40-kDa band could be detected after incubation with the H3 antibody on both membranes.

We tested two antibodies in parallel to detect NR2E3 expression by western blot analysis: a mouse monoclonal antibody (clone H7223) directed to amino acids 2–45 of NR2E3 and a rabbit polyclonal directed to the whole NR2E3 protein. Immunoblotting of the cells that were transfected with the WT and G56R plasmids detected a full-length 45-kDa NR2E3 protein in both HEK293 (Figure 5A,B) and COS7 cell lines, as expected. By contrast, immunoblotting of the G56R-CRISPR2-expressing cells only revealed a small protein that corresponded to the predicted ~16 kDa size of the truncated NR2E3 (Figure 5A; 55-kDa tubulin band served as a loading control). Interestingly, only the mouse monoclonal antibody detected the 16-kDa band, whose epitope remains intact in G56R-CRISPR2, and not by the polyclonal antibody (Figure 5B; a 42-kDa actin band served as a loading control), which suggested that the polyclonal antibody has a higher affinity for the C-terminal part of the protein that was lost in the G56R-CRISPR2 clone.

Following the western blot results, we only used the mouse monoclonal NR2E3 antibody for IF analyses to ensure that we would detect truncated NR2E3 expression. Following the transfection of HEK293 and COS7 (Figure 5C) cells with the three constructs, we detected protein expression in all cases, but the cellular localization differed strikingly between the three. WT NR2E3 showed a mixed perinuclear and nuclear localization (Figure 5C, left), whereas G56R NR2E3 preferentially showed a nuclear localization (Figure 5C, middle). We quantified three independent IF experiments to evaluate the different localization between WT and G56R. The analysis demonstrated that 89 ± 1.9% of WT-transfected cells showed a perinuclear NR2E3 localization, as compared to 32 ± 4.8% of G56R-transfected cells (Figure 5D). Conversely, 10.8 ± 2% of WT-transfected cells showed a purely nuclear NR2E3 localization as compared to 68 ± 4.8% of G56R-expressing cells (Figure 5E). These differences were statistically significant in both cases (*p* < 0.05).

Furthermore, and in direct contrast to WT and G56R, the expression of the truncated G56R-CRISPR2 NR2E3 protein was much weaker and localized throughout the cytosol (Figure 5C, right). The cytosolic and nuclear fractions of HEK293 (Figure 5E) and COS7 cell lines that were transfected with the WT, G56R, and G56R-CRISPR2 plasmids were separated by differential centrifugation and analyzed by western blot analysis in order to confirm this localization profile. Consistent with the (peri)nuclear labelling detected by IF, WT and G56R proteins could be detected in both fractions, whereas the 16-kDa G56R-CRISPR2 protein was exclusively localized to the cytosolic fraction; no band was detected in the nuclear fraction. To verify the purity of the fractions, the membranes were hybridized with an anti-tubulin antibody, as a cytosolic marker, and an anti-histone H3 antibody, as a nuclear marker. A 55-kDa tubulin was only detected in the cytosolic fraction and an ~15-kDa H3 in the nuclear fraction; a non-specific ~40-kDa band that was associated with the anti-H3 antibody was seen in both fractions.

Taken together, the overexpression of allele-specific *NR2E3* knockout results in a truncated and mis-localized protein in cell lines.

### 2.6. Assessing the Retinal Organoid Differentiation Potential of a G56R-CRISPR iPSC Line

**Figure 6:**
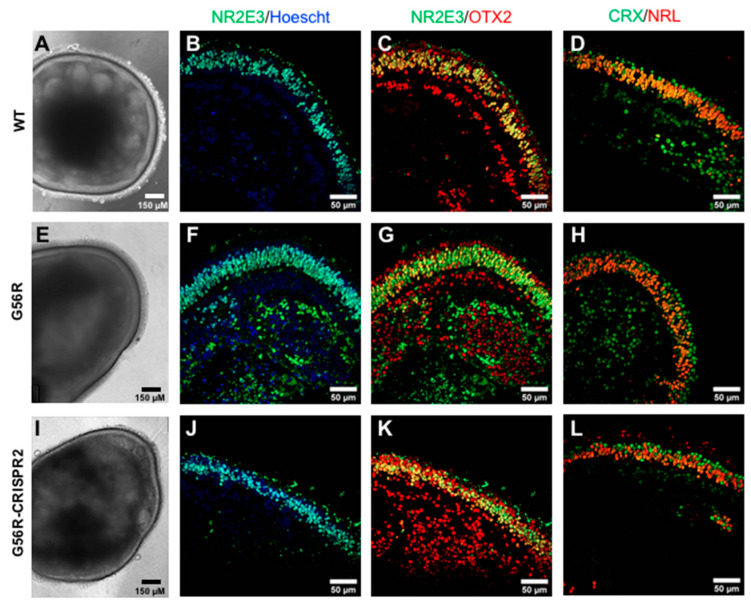
Retinal differentiation potential following *NR2E3* allele knockout. Bright-field images of WT (**A**), G56R (**E**), and G56R-CRISPR2 (**I**) iPSC-derived retinal organoids at 180 days of differentiation. IF analysis of NR2E3 expression (in green) in WT (**B**), G56R (**F**), and G56R-CRISPR2 (**J**) retinal organoids; nuclei labelled in blue. NR2E3 expression is localized to the photoreceptor nuclei, which form an outer layer, corresponding to the outer nuclear layer (ONL), in the organoid. Colocalization studies of NR2E3 (in green) and OTX2 (in red) expression in WT (**C**), G56R (**G**) and G56R-CRISPR2 (**K**) retinal organoids. NR2E3 is restricted to the rod nuclei whereas OTX2 also labels the more peripheral cone nuclei, as well as retinal cells situated on the inner side of the ONL. Colocalization studies of CRX (in green) and NRL (in red) expression in WT (**D**), G56R (**H**), and G56R-CRISPR2 (**L**) retinal organoids. NRL expression is restricted to rod nuclei, whereas CRX also labels the cone nuclei situated on the outer rim of the organoids.

The final part of this study was to investigate whether the genome-edited iPSCs could differentiate into retinal organoids. To this end, we used a previously reported two-dimensional (2D)–three-dimensional (3D) retinal differentiation protocol [35]. Briefly, iPSCs were cultured in 2D feeder-free conditions until neuroepithelial structures with a typical mushroom morphology and a peripheral lamination emerged. At 28 days post-differentiation, we manually excised the organoid structures and individually transferred them to free-floating culture. All three iPSC lines, WT (Figure 6A), G56R (Figure 6E), and G56R-CRISPR2 (Figure 6I), were differentiated into retinal organoids. At 180 days of differentiation, bright-field microscopy showed a characteristic lamination in the organoids that corresponded to the retinal outer nuclear layer (ONL). Furthermore, the retinal organoids presented with a typical brush border, which corresponded to photoreceptor outer segments.

We analyzed the expression of NR2E3 by IF analysis using the mouse monoclonal antibody. NR2E3 was mainly expressed in the ONL of WT (Figure 6B), G56R (Figure 6F), and G56R-CRISPR2 (Figure 6J) organoids. Colocalization studies of NR2E3 and OTX2 expression showed that NR2E3 was restricted to rod nuclei of WT (Figure 6C), G56R (Figure 6G), and G56R-CRISPR2 (Figure 6K) organoids, whereas OTX2 also labelled cone nuclei (the OTX2-positive/NR2E3-negative nuclei situated towards the outer rim). Furthermore, because OTX2 is an early developmental marker expressed prior to photoreceptor differentiation, its expression was also detected in an inner layer of nuclei that likely represents retinal progenitor or bipolar cells [36]. Similar results were obtained by colocalization studies of the two partners of NR2E3, CRX, and NRL, in the WT (Figure 6D), G56R (Figure 6H), and G56R-CRISPR2 (Figure 6L) organoids, whereby NRL expression was restricted to the rod nuclei, whereas CRX also labelled cone nuclei (CRX-positive/NRL-negative nuclei in the outer rim).

In conclusion, we showed that allele-specific *NR2E3* knockout does not affect the retinal differentiation potential of iPSCs or NR2E3 expression.

## 3. Discussion

The G56R mutation in NR2E3 is the second most common mutation causing adRP, a disorder for which there is currently no cure [3]. Genome editing offers a host of therapeutic options for IRDs in terms of gene and cell therapy [37], but the feasibility of clinical translation may be variable. For example, gene correction requires HDR and, thus, may not reach therapeutic efficiency for gene therapy, due to the post-mitotic nature of photoreceptors, but holds promise for cell therapy. By contrast, a HDR-independent strategy that involves specific knockout of a mutant allele could be a potentially efficient gene therapy approach. This would be particularly pertinent in the case of G56R, as this mutation exclusively causes all *NR2E3*-associated adRP forms [4]. Therefore, a single therapeutic product could potentially treat all patients, which renders it economically attractive. Here, for the first time, we developed a CRISPR/Cas9-mediated allele knockout strategy to treat *NR2E3*-associated adRP. The efficiency and specificity of this approach suggests that it could be a promising future treatment for *NR2E3* patients.

Of the two allele-specific knockout systems that we designed, the most optimal was the one that was made up of the gRNA1 molecule spanning the G56R mutational site and adjacent to a NGG PAM sequence that can be recognized by eSpCas9. The combination of the allele specificity of the gRNA and the enhanced specificity feature of eSpCas9 resulted in specific targeting of the mutant allele in 75% of the surviving iPSC clones; in the remaining 25%, no genome editing events took place. Importantly, the wild type allele was never targeted in the surviving clones, and no off-target events were detected. The edited clones contained 1- or 2-bp indels within exon 2 of *NR2E3*, which gave rise to a PTC. It has been established that NMD takes place when a PTC emerges ≥50–55 nt upstream of the last exon-exon junction [38,39]. In our case, the PTCs were located >800 nt upstream of the last exon 7 to exon 8 junction. Therefore, it is highly likely that the mRNA that is transcribed from the edited DNA would undergo NMD, before producing a mutant protein, which corresponds to an effective knockout. In this way, we can exclude the risk that the truncated protein further interferes with the WT NR2E3 allele.

Nevertheless, it has been reported that, after CRISPR/Cas-mediated knockout, mRNAs can escape NMD. This can happen through different mechanisms, such as exon skipping, the use of alternative initiation codons, or conversion of mRNAs with a PTC into protein-coding molecules [40]. In addition, a truncated protein is sometimes detectable [41]. We overexpressed the G56R-CRISPR cDNA in an exogenous system wherein NMD would not occur in order to investigate what would happen at the protein level in case the mRNA escaped NMD [42]. Under these conditions, we detected a truncated 16-kDa protein from the G56R-CRISPR cDNA, which corresponded to the predicted truncation of NR2E3 within the DBD. Furthermore, this truncated protein showed a defective localization as compared to WT NR2E3 and even when compared to the mutated G56R NR2E3 protein. More specifically, G56R-CRISPR2 NR2E3 expression was diffused throughout the cytosol, whereas WT NR2E3 expression was restricted to the (peri)nuclear region. Therefore, even if G56R-CRISPR escaped NMD in the photoreceptors, its altered structure and mis-localization would most likely lead to its degradation [43].

The removal of a nuclear localization signal (NLS) would be a plausible explanation for the cytoplasmic localization of G56R-CRISPR NR2E3. An in silico search of the 410 aa NR2E3 protein sequence (NP_055064.1) for an NLS using the database cNLS mapper [44], identified a putative, but low-score, bipartite NLS at aa position 69 in the DBD. Consistently, other members of the Nuclear Receptor family are known to contain NLS in the DBD, in the hinge region, or even the LBD [45,46]. The predicted NLS in the NR2E3 DBD is missing from the truncated G56R-CRISPR proteins, which could account for the lack of nuclear targeting. By contrast, the NLS is unaffected by the G56R variant, which is consistent with the nuclear localization of this mutant protein.

What was particularly interesting from this study was the mixed nuclear and perinuclear staining for WT NR2E3, which was a striking contrast from the predominant nuclear staining of the G56R mutant. These results could suggest that NR2E3 shuttles between the nucleus and the cytoplasm to accomplish its roles in the cell. This would not be surprising, as many nuclear receptors are known to reside in the cytoplasm [46], but, until now, it has not been described for NR2E3. Different mechanisms have been reported for the export of nuclear receptors, including nuclear export signals (NES), protein–protein interactions, and posttranslational modifications [47]. The DBD has been reported to act as a NES for many nuclear receptors, such as the Retinoic X receptor, which belongs to the same subfamily as NR2E3 [47]. Thus, we could hypothesize that this is also the case for NR2E3 and that the G56R mutation, which is in the DBD, could directly affect its nuclear export ability. We also screened the NR2E3 sequence for a potential NES while using the LocNES prediction tool [48]. NES were predicted from aa positions 359 to 381 within the LBD, with the highest scoring NES at position 366–380. However, it is difficult to understand how G56R could disrupt this C-terminal signal. 

A previous study reported the subcellular localization of a panel of *NR2E3* variants, including G56R [16]. The studied variants were scored according to a nuclear or cytoplasmic localization and, consistent with our data, G56R was reported to be nuclear. Although there was no strict correlation between the position (DBD versus LBD) of the variants studied and the resulting localization profile, it can be said that the majority of variants resulting in a nuclear localization were situated upstream of aa position 232, and the majority resulting in a cytoplasmic (or mixed) localization were situated downstream (i.e., in the LBD). This could be consistent with the predicted presence of the NES with the LBD of NR2E3 at aa position 366–380. In contrast to our observations, this previous study described a nuclear localization for wild type NR2E3. This is surprising, as there is no doubt as to the perinuclear NR2E3 staining in our study. The cell lines that were used in the two studies were similar (COS1 versus COS7 here), so it is unlikely that this underlies the differences observed. However, the previous study [16] used a pcDNA4 His/Max expression system, which fused a 4-kDa Xpress epitope to the NR2E3 protein. Thus, it is possible that the tag partially interfered with the intracellular trafficking/localization of NR2E3.

We conducted a preliminary iPSC-derived retinal organoid study to further investigate the effect of allele-specific knockout of G56R NR2E3 in a native environment and, more specifically, on the retinal differentiation potential. We show, for the first time, that G56R NR2E3 iPSCs can differentiate into retinal organoids. This is consistent with the fact that G56R is associated to progressive RP, which is not a congenital disorder. We also show that G56R-CRISPR iPSCs retain the potential for retinal differentiation. Although, a thorough comparison of the phenotypic differences between WT, G56R, and G56R-CRISPR organoids was beyond the scope of this study, we observed that G56R-CRISPR2 organoids expressed NR2E3 in a lamination that was indistinguishable from WT. This suggests that *NR2E3* allele-specific ablation would mimic the situation of ESCS loss-of-function mutation carriers who are asymptomatic, and that clinical translation of our CRISPR/Cas-mediated knockout strategy would not have negative effects on the human retina.

Along this line, a previous study reported the generation of iPSC-derived retinal organoids from homozygous carriers of *NR2E3* mutations [49]. In non-laminated retinal organoids, the authors detected S cones in patient organoids. By contrast, no S cones were detected WT organoids at the same differentiation timepoint, which suggested that the homozygous *NR2E3* organoids mimicked the ESCS phenotype. iPSC-derived retinal organoids from heterozygous carriers were not generated in this previous study. Thus, the future characterization of G56R-CRISPR organoids in comparison to WT, in addition to validating our therapeutic strategy, will also indirectly provide information on the phenotype of *NR2E3* ESCS carriers. Furthermore, although the phenotype of *NR2E3* carriers suggest that a single allele will be sufficient for correct photoreceptor function, we do not yet exclude the necessity of coupling mutant allele knockout to *NR2E3* gene supplementation. The G56R and G56R-CRISPR organoids will also provide the opportunity to directly address this question in a pertinent setting.

Nevertheless, allele-specific knockout approaches via CRISPR/Cas have been successfully used in vivo in the case of certain IRDs, thus we are relatively confident that this would also be the case for the *NR2E3* approach described herein. The recurrent P23H mutation in rhodopsin was one of the first mutations to have been successfully targeted by such a strategy. Proof-of-concept studies in P23H mouse models demonstrated reduced expression of the mutant protein [50], as well as retinal preservation and improved retinal function [51,52]. Another highly pertinent target is the recurrent c.2991+1655A>G variant in intron 26 of the gene *CEP290* [53], which was associated with autosomal recessive Leber congenital amaurosis. Allele-specific knockout of the intronic variant restored WT *CEP290* splicing in animal models [54,55], and it has led to the first-in-humans Phase 1/2 clinical trial for CRISPR/Cas genome editing of a genetic disorder (NCT03872479). This suggests that clinical translation of a similar strategy for NR2E3 G56R may not be a pipedream.

Allele-specific knockdown (as opposed to knockout) can also be accomplished using antisense oligonucleotide (AON)-based approaches, which have the advantage of being considered to be safer, as the host DNA is not targeted, but also the drawback of requiring repeat administration due to AON turnover. The *CEP290* intron 26 variant has served as the target for AON-based proof-of-concept studies [56,57], which have also led to Phase 1/2 clinical trials (NCT03140969). Along this line, an AON approach that was designed to specifically target the G56R variant was previously reported for *NR2E3* [58]. Encouragingly, a reduction in both G56R *NR2E3* mRNA and protein levels was achieved. However, a reduction in WT mRNA and protein levels was also observed, indicating a lack of target specificity for the mutant allele. In this regard, CRISPR/Cas-based approaches hold significant advantages over AONs, because, in addition to only requiring a single administration, their high specificity allows allele discrimination to the level of a single nucleotide change, as shown in this study.

Taken together, here we report the first proof-of-concept study demonstrating the high specificity and efficiency of allele-specific knockout of the NR2E3 G56R mutant allele while using CRISPR/Cas9. This clinically feasible approach represents a promising strategy to treat all patients who develop *NR2E3*-associated adRP.

## 4. Materials and Methods

### 4.1. Skin Biopsy and Fibroblast Culture

The skin biopsy of the *NR2E3*-associated adRP patient volunteer was performed at the National Reference Center for Inherited Sensory Diseases (Maolya, Montpellier, France) following signed informed consent. The biomedical research study (project identification code 2014-A00549-38) was approved on the 13 June 2014 under the authorization number 140549B-62 by the French National Agency for the Safety of Medicines and Health Products (ANSM). The biopsy and emerging fibroblasts were cultured in AmnioMAX C100 basal media with L-glutamine (Gibco, Thermo Fisher Scientific, Villebon sur Yvette, France) that contained 10% decomplemented foetal bovine serum (FBS; Gibco), 1% penicillin-streptomycin amphotericin B (Gibco), and 2% AmnioMax-C100 supplement (Gibco). The fibroblasts were passaged using 0.25%Trypsin (Gibco) and then cryo-preserved in FBS that was supplemented with 10% DMSO (Sigma Aldrich, St. Quentin Fallavier, France).

### 4.2. iPSC Reprogramming and Culture

Fibroblasts were reprogrammed, as previously described using the CytoTune-iPS 2.0 Sendai Reprogramming Kit (Thermo Fisher Scientific, Villebon sur Yvette, France) [28]. At day seven post-transduction, the transduced fibroblasts were passaged onto Matrigel-coated plates (Corning hESC-Qualified Matrix, Dominique Dutscher, Brumath, France) and, the following day, the medium changed to TeSR-E7 Basal Medium (STEMCELL Technologies, Grenoble, France). The emerging iPSC colonies were mechanically transferred into supplemented Essential (E) 8 Medium (Gibco) and subsequently passaged using Versene Solution (Gibco).

### 4.3. DNA Sequencing

Genomic DNA was isolated from fibroblasts using the DNeasy Blood & Tissue Kit (Qiagen, Courtaboeuf, France) and then amplified by PCR using AmpliTaq Gold™ 360 Master Mix (Applied Biosystems, Thermo Fisher Scientific, Villebon sur Yvette, France) and *NR2E3*-specific primers (Supplementary Table S1) on a Veriti thermocycler (Applied Biosystems). The amplicons were cleaned with the ExoSAP-IT PCR Clean-up kit (GE Healthcare, Velizy Villacoublay, France) and sequenced using the BigDye Terminator Cycle Sequencing Ready Reaction kit V3.1 (Applied Biosystems). The analyses were performed on an Applied Biosystems 3130xL genetic analyzer.

### 4.4. RT-PCR and qPCR

The total RNA from fibroblasts and iPSCs was extracted with the RNeasy Mini Kit (Qiagen). cDNA was synthesized from 500 ng of RNA with the SuperScript III First-Strand Synthesis System using random hexamers (Life Technologies, Thermo Fisher Scientific, Villebon sur Yvette, France). The cDNA was diluted 1:10 and 2 μL used per PCR reaction (10 µL total). The qPCR reaction was performed using the FastStart SYBR Green I Master mix and the LightCycler 480 II thermal cycler (Roche, Meylan, France). The gene expression was normalized to *GAPDH* expression. Primers sequences were as previously reported [28].

### 4.5. Chromosome Integrity Analyses

The preparation of the G56R iPSCs for chromosome spreading was performed as previously described [59]. Twenty metaphase spreads were counted, and karyotype analysis was performed by the Chromostem facility (CHU Montpellier, France). Analysis of the G56R-CRISPR iPSCs was performed while using the iCS-digital TM PSC test by Stem Genomics (Montpellier, France).

### 4.6. Embryoid Body and Retinal Organoid Differentiation

The iPSCs were differentiated into EBs, as previously described, without modification [29]. iPSCs were differentiated into retinal organoids based on a previously described protocol [60,61]. Briefly, iPSCs were cultured in E8 on Matrigel dishes to 70–80% confluency, and the medium was changed to E6; this was defined as day 0 of differentiation. At day 2, 1% N2 supplement was added. On day 28, neuro-retinal organoids were excised with a scalpel and then transferred to ultra-low attachment 24-well plates for individual free-floating culture and they were maintained in DMEM/F12, 1:1, medium with Glutamax (Gibco) containing 1% MEM nonessential amino acids (Gibco), 1% Glutamax (Gibco), 1% B27 supplement (Gibco), 10 units/mL Penicillin, and 10 µg/mL Streptomycin (Gibco), and then supplemented with 10 ng/mL of animal-free recombinant human FGF2 (Miltenyi Biotec, Paris, France). At day 35, to keep laminated neuroretinal structures, FGF2 was removed from the medium and 10% FBS was added [35,62]. At day 85, the B27 supplement was replaced by B27 supplement without vitamin A (Gibco). During the entire differentiation protocol, the medium was changed three times per week; for the free-floating culture, half of the medium was refreshed three times per week.

### 4.7. Immunofluorescence Staining

iPSCs and embryoid bodies were fixed in 4% PFA for 20 min at room temperature, permeabilized with 0.1% Triton X-100 (Sigma-Aldrich, St. Quentin Fallavier, France), and blocked with 5% BSA and 10% donkey serum (Millipore, Saint Quentin en Yvelines, France) for 1 h. Retinal organoids were collected at day 180 of retinal maturation, washed twice in PBS, fixed in 4% PFA for 20 min at 4 °C, incubated in 20% sucrose overnight at 4 °C, embedded in Tissue freezing medium (Microm Microtech, Brignais, France), and then frozen on dry ice. Embedded organoids were cut into 10 µm cryosections using a Leica CM3050 cryostat and collected on Superfrost Plus glass slides (Thermo Fisher Scientific, Villebon sur Yvette, France). The primary and secondary antibodies are listed in Supplementary Table S2 and Table S3, respectively. The primary antibodies were incubated overnight at 4 °C; an overnight incubation without primary antibody was used for the negative control. The secondary antibodies and 0.2 µg/mL bisBenzimide (Sigma-Aldrich) were incubated for 1 h at room temperature. The samples were observed using a Zeiss ApoTome 2 Upright wide-field microscope or a Confocal Zeiss LSM700 microscope.

### 4.8. Design of gRNAs and Plasmid Construction

CCTOP (https://crispr.cos.uni-heidelberg.de), CRISPOR (http://crispor.tefor.net), and MIT CRISPR online design tool (https://zlab.bio/guide-design-resources) were used for designing gRNA1 for eSpCas9, and CCTOP and CRISPOR for designing gRNA2 for VQR Cas9. We only retained gRNAs that spanned the mutational site. The plasmids used for cloning the gRNAs were eSpCas9(1.1) (#79145; Addgene), and p458 VQR (#101727, Addgene). The complementary oligonucleotides for each gRNA were produced by Eurogentec (Angers, France) (Supplementary Table S1). The oligonucleotide pairs were annealed after 5 min of denaturation at 95 °C and slow-cooled to 50 °C (ramp rate 0.2 °C/s), incubated at 50 °C for 10 min, and then further cooled to 4 °C (ramp rate 1 °C/s). The corresponding plasmids were digested with the *Bbs*I restriction enzyme prior to ligation using the T4 DNA ligase (Promega, Charbonnières les Bains, France) overnight at 4 °C, according to the manufacturer’s instructions.

### 4.9. iPSC Transfection and FACS

iPSC transfection and sorting was performed, as previously described [33]. iPSCs were cultured in mTeSR1 medium (STEMCELL Technologies, Grenoble, France) pre- and post-transfection. Large-scale and high-purity plasmids were prepared using the Qiagen EndoFree Plasmid Maxi kit. The iPSCs were dissociated with Accutase (STEMCELL Technologies), and 1.5 × 10^6^ cells were electroporated with 5 µg of plasmid DNA while using the Amaxa nucleofector system (Lonza, Levallois-Perret, France). Following transfection, cells were resuspended in mTeSR1 medium that was supplemented with 10 mM Rho-associated kinase (ROCK) inhibitor Y-27632 (StemMACS; Miltenyi Biotec, Paris, France) and then seeded in 24-well plates. Forty-eight hours post-transfection, EGFP-positive cells were single cell-sorted by FACS (FACSAria III, Becton Dickinson, San Jose, CA, USA) into 96-well plates. Two to three weeks post-electroporation, the surviving colonies were manually picked and expanded for culture and screening.

### 4.10. T7 Endonuclease I Assay

The T7EI assay was performed in transfected iPSCs, as described in [33]. The region surrounding the mutation was amplified with specific primers (Supplementary Table S1) by the high-fidelity TaKaRa polymerase (Thermo Fisher Scientific). The PCR products were then denatured at 95 °C for 5 min and gradually reannealed at 95–85 °C (ramp rate of –2 °C/s), followed by 85–25 °C (ramp rate of −0.3°C/s) to allow the formation of heteroduplexes. The reannealed amplicons were then incubated with T7EI (New England Biolabs, Evry, France) at 37 °C for 1 h and the reaction stopped by the addition of Proteinase K (Qiagen) at 37 °C for 5 min. The digested products were analyzed by 1% ultrapure agarose gel electrophoresis.

### 4.11. Off-Target Analysis

The possible off-targets regions were predicted by the CRISPOR software, which used the MIT and CFD scores. The top five exonic off-targets for each of the scores (10 in total) were selected for analysis. Primer Blast was used to design the primers flanking the predicted regions (Supplementary Table S1). PCR amplification and sequencing was performed on the genomic DNA isolated from the G56R-CRISPR iPSC lines and a WT iPSC line, as described above.

### 4.12. Mutagenesis and NR2E3 cDNA Plasmid Construction

The *NR2E3* cDNA was isolated from the Clontech Human Retina QUICK-Clone cDNA pool (TaKaRa Bio Europe, Saint Germain en Laye, France) while using specific primers (Supplementary Table S1), and the amplicon was cloned into the pGEM-T Easy vector system (Promega), according to the manufacturer’s instructions. Mutagenesis was performed using the QuickChange Site-Directed Mutagenesis Kit (Agilent Technologies, Montpellier, France) to introduce the c.166G>A (G56R) and c.173_174delAC (G56R-CRISPR2) mutations. The WT and mutated NR2E3 sequences were verified by Sanger sequencing prior to sub-cloning into the pcDNA3 expression system (Invitrogen; kindly provided by Dr. J. Deveaux, INM). For sub-cloning, the pGEM-T Easy constructs and pcDNA3 plasmid were both digested by the restriction enzyme *Eco*RI. The pcDNA3 plasmid was dephosphorylated using the FastAP thermosensitive alkaline phosphatase (Thermo Fisher Scientific), and both the vector and inserts were purified using the NucleoSpin Gel and PCR Clean-up kit (Macherey-Nagel, Hoerdt, France) prior to ligation and subsequent transformation in One Shot TOP10 chemically competent *E. coli* (Thermo Fisher Scientific, Villebon sur Yvette, France).

### 4.13. HEK293 and COS7 Culture and Transfection

The HEK293 (293T; ATCC CRL-3216) and COS7 (ATCC CRL-1651) cells were cultured in DMEM (Gibco) that was supplemented with 10% FBS. For IF studies, cells were seeded on poly-lysine coated coverslips in 24-well plates and for western blot analysis in 12-well plates at a density of 9 × 10^5^ cells/cm^2^ (HEK293) or 5 × 10^5^ cells/cm^2^ (COS-7). For the differential centrifugation studies, both of the cell lines were seeded in six-well plates at a density of 6 × 10^5^ cells/cm^2^. Twenty-four h post-seeding, cells were transfected using lipofectamine 3000 (Thermo Fisher Scientific) and 500 ng (24-well), 1 µg (12-well), or 2.5 µg (6-well) of plasmid DNA. For IF studies, 48 h post-transfection, cells were fixed in 4% PFA for 30 min at 4 °C, permeabilized with 0.3% Triton X-100 (Sigma-Aldrich), and blocked with 10% BSA and 10% donkey serum for 1 h. The antibodies were diluted in antibody dilution buffer (0.1% Triton X-100, 10% BSA, and 1% donkey serum) and IF performed, as described for iPSCs.

### 4.14. Western Blot Analyses

HEK293 and COS7 cells were washed in PBS and then lysed in RIPA lysis buffer containing the complete protease inhibitor cocktail (Roche, Meylan, France). The cell lysates were centrifuged at 20,000× *g* for 15 min, and the resulting supernatant was quantified using the BCA protein assay kit. The equivalent of 7.5 µg of protein was mixed with Laemmli sample buffer and 1 µL of β-mercaptoethanol in a volume of 25 µL. The samples were heated at 95 °C for 5 min and then loaded onto an AnyKD precast MiniProtean TGX Stain Free Gel. After electrophoresis, the proteins were transferred to a nitrocellulose membrane using a Trans-Blot TurboTM Transfer Pack and System. Following transfer, the membranes were blocked with 0.5% Tween-PBS in 5% skim milk for 1 h at room temperature and then incubated with the primary antibodies (Supplementary Table S2; the anti-histone H3 antibody was kindly provided by Dr. R. Feil, IGMM) diluted in blocking solution, overnight at 4 °C. The membranes were then washed three times for 15 min in 0.5% Tween-PBS and incubated with the secondary antibody (Supplementary Table S3) in blocking solution for 45 min at room temperature. Following three washes, the membranes were dried and visualized using an Odyssey CLx imager (Li-Cor Biotechnology, Bad Homburg, Germany).

### 4.15. Differential Centrifugation

The cell fractions were prepared, as previously described [63], and then adapted to cells in a six-well format. Briefly, the cells were washed with PBS, harvested by scraping, and transferred to a 1.5 mL tube. Cells were pelleted by centrifugation 30 s on a bench-top centrifuge. The pellet was resuspended in 108 µL ice-cold 0.1% NP-40 in PBS and triturated five times using a 200 µL micropipette, and then re-centrifuged for 30 s. For the cytosolic fraction, 36 µL of the supernatant was collected and mixed with 12 µL of 4× Laemmli sample buffer. For the nuclear fraction, the remaining material was re-centrifuged for 30 s and the pellet was washed once with 0.1% NP-40 in PBS, resuspended in 110 µL 1× Laemmli sample buffer, and sonicated twice for 5 s at level 2 using a Sonicator ultrasonic processor XL (Misonix, Inc., Farmingdale, NY, USA). After the addition of β-mercaptoethanol, the samples were heated at 95 °C for 5 min, loaded onto an AnyKD precast MiniProtean TGX Stain Free Gel, and processed, as described above.

## 5. Patents

V. Kalatzis and M. Diakatou. Allele-specific genome editing of the *NR2E3* mutation G56R. EP21305224.4. Filed 25 February 2021.

## Data Availability

No new data were created or analyzed in this study. Data sharing is not applicable to this article.

## Supplementary Materials

The following are available online at https://www.mdpi.com/1422-0067/22/5/2607/s1.

## Abbreviations

aa	Amino Acid(s)
ad	Autosomal Dominant
AFP	Alpha Fetal Protein
Cas	CRISPR-associated
COS7	CV-1 Origin Sv40
CNV	Copy Number Variant
CRISPR	Clustered Regularly Interspaced Short Palindromic Repeats
CRX	Cone-Rod Homeobox
DBD	DNA Binding Domain
DSB	Double-Strand Break
EGFP	Enhanced Green Fluorescent Protein
ESCS	Enhanced S Cone Syndrome
GFAP	Glial Fibrillary Acidic Protein
gRNA	Guide RNA
HDR	Homologous-Directed Repair
HEK293	Human Embryonic Kidney
indels	Insertions/deletions
iPSC	induced Pluripotent Stem Cell
IRD	Inherited Retinal Dystrophies
KLF4	Kruppel-Like Factor
LBD	Ligand Binding Domain
LIN28A	Lin-28 Homolog A
NANOG	Nanog Homeobox
NES	Nuclear Export Signal
NHEJ	Non-Homologous End Joining
NLS	Nuclear Localization Signal
NMD	Nonsense-Mediated Decay
NR1D1	Nuclear Receptor Subfamily 1 Group D Member 1
NR2E3	Nuclear Receptor Subfamily 2 Group E Member
NRL	Neural Retina Leucine Zipper
OCT3/4	Octamer Binding Transcription Factor
OTX2	Orthodenticle Homeobox 2
PAM	Protospacer Adjacent Motif
PTC	Premature Termination Codon
RP	Retinitis Pigmentosa
SeV	Sendai Virus
SMA	Smooth Muscle Actin
SOX2	SRY-Box Transcription Factor 2
Sp	*Streptococcus Pyogenes*
T7E1	T7 Endonuclease 1
WT	Wild type
