# Supplementary material for: Allele-Specific Knockout by CRISPR/Cas to Treat Autosomal Dominant Retinitis Pigmentosa Caused by the G56R Mutation in NR2E3
Source: Int J Mol Sci. 2021 Mar 5;22(5):2607. doi: 10.3390/ijms22052607 (PMC7961898; doi:10.3390/ijms22052607)
Supplement: Supplementary file 1 [file ijms-22-02607-s001.pdf]

Table S1. Sequences of the primers used in this study.

Target	Forward Primer	Reverse Primer
NR2E3		
cDNA amplification	5′ GG GCA CAG AGA GAC AGA GGT TC 3′	5′ GCT AGC ATT TCA CCT CCA CCC CCA 3′
Exon 2 sequencing	5′ GTT CAA ATG CGG GTG AGC 3′	5′ GTC AGT GTC CCT CCC ATG C 3′
T7E1 assay	5′ GCC TGA GGA CTG GGA AAG G 3′	5′ CAC CGC ACT CAC CTG TAG AT 3′
**gRNA oligonucleotides**		
gRNA1	5′ cacc AGC AGC AGC AGG AAG CAC TA 3′	5′ aaac TAG TGC TTC CTG CTG CTGC T 3′
gRNA2	5′ cacc GTG CGG AGA CAG CAG CAG CA 3′	5′ aaac TGC TGC TGC TGT CTC CGC AC 3′
**Off-target genes**		
ESRRB	5′ CAT CAA GAC TGA GCC GTC CA 3′	5′ ACC ACG CAC CTT GGA TAG TC 3′
GPR135 GD	5′ ACA ACT GGA TTT TTG CGG GC 3′	5′ TGG ACC GTT ACT GCG CTA TC 3′
SOX17	5′ TTT TCG AAC GAC CGG GTGA T 3′	5′ ATC TCA GGC TTG CAC ACG AA 3′
RP1-37C10.3	5′ TGA CAA GCA AAC CCC GTG TA 3′	5′ CCT ACA GAA GGC TGA CGG AC 3′
NPEPL1/STX16-NPEPL1	5′ CTC CCT GGA TCC ACC CCT TA 3′	5′ TTG TCT CGG TCC TAG GGA GG 3′
TSPAN4	5′ GAC TTG GGA CCA GAA CCC TG 3′	5′ CTG GTA AAT CCA CAC CCG CT 3′
SLC39A4	5′ GCA GGC AGG GTC TCC ATA TC 3′	5′ CTC CTC TTC CTG CTG CAC AA 3′
TBX5-AS1	5′ TCC CAC CGA ATG CTC TCA AC 3′	5′ AAC AGG AGG CGT TGG GTT AG 3′
FAM69B/SNHG7	5′ GCG GGA CAA GGA TGT AAC CA 3′	5′ CAG GTC GGC CAT CTT GAA GT 3′
PDLIM7	5′ CAC CCC AAA TGC AAA CCA GG 3′	5′ GTC TAC TCC TGA GGC TCC CA 3′
**Mutagenesis primers**		
WT to G56R	5′ GAG ACA GCA GCA GCA GGA AGC ACT ATG G 3′	5′ GCC ATA GTG CTT CCT GCT GCT GCT GTC TC 3′
G56R to G56R-CRISPR2	5′ GCA GCA GCA GGA AGC TAT GGC ATC TAT GCC 3′	5′ GGC ATA GAT GCC ATA GCT TCC TGC TGC TGC 3′

Table S2. List of the primary antibodies used in this study.

Antibodies	Host	Dilution	Company; Catalogue Number	Location/Distributor
**IF studies**				
Pluripotency markers				
NANOG	rabbit	1/200	Abcam; ab21624	Paris, France
OCT3/4	mouse	1/200	Santa Cruz Biotechnology; sc-5279	Heidelberg, Germany
SOX2	rabbit	1/200	ThermoFisher Scientific; 48–1400	Villebon sur Yvette, France
Differentiation markers				
AFP	mouse	1/200	Sigma-Aldrich; WH0000174M1M; clone 1G7	St. Quentin Fallavier, France
SMA	mouse	1/200	Dako; M0851	Les Ulis, France
Nestin	mouse	1/200	Novus Biologicals; NBP1–92717	Lille, France
GFAP	rabbit	1/200	Dako; Z0334	Les Ulis, France
Retinal Organoids				
NR2E3	mouse	1/150	R&D Systems; PP-H7223-00; clone H7223	Bio-Techne SAS, Noyal Châtillon sur Seiche, France
OTX2	goat	1/100	R&D Systems; AF1979	Bio-Techne SAS
CRX	mouse	1/2000	Abnova; H00001406-M02	Paris, France
NRL	goat	1/150	R&D Systems; AF2945	Bio-Techne SAS
**Western blot analyses**				
NR2E3 (monoclonal)	mouse	1/2000	R&D Systems; PP-H7223-00; clone H7223	Bio-Techne SAS
NR2E3 (polyclonal)	rabbit	1/2000	Proteintech; 14246-1-AP	Manchester, UK
β-tubulin	rabbit	1/5000	Cell Signaling Technology; 2128	Ozyme, Saint Cyr L'Ecole, France
β-actin	mouse	1/5000	Sigma Aldrich; A5316	St. Quentin Fallavier, France
Histone H3	mouse	1/1000	Millipore; 06-755	St. Quentin en Yvelines, France

Table S3. List of secondary antibodies used in this study.

Antibodies	Host	Dilution	Company; Catalogue number	Distributor/Location
**IF studies**				
Alexa Fluor 488-conjugated anti-mouse	donkey	1/500	Jackson ImmunoResearch; 715-546-151	Cambridgeshire, UK
Alexa Fluor 647-conjugated anti-rabbit	donkey	1/500	Jackson ImmunoResearch; 711-606-152	Cambridgeshire, UK
Alexa Fluor® 594-congugated anti-goat	donkey	1/500	Jackson ImmunoResearch; 705-586-147	Cambridgeshire, UK
**Western blot analyses**				
IRDye 800CW anti-Rabbit	donkey	1/20 000	Li-Cor Biotechnology; 926-3221	Bad Homburg, Germany
IRDye 680RD anti-mouse	goat	1/20 000	Li-Cor Biotechnology; 926-68070	Bad Homburg, Germany

```
hNR2E3 gene         GCCGCGTGTGCGGAGACAGCAGCAGCGGGAAGCACTATGGCATCTATGCC
CRISPR2 colony 1    GCCGCGTGTGCGGAGACAGCAGCAGCAGGAAGC--TATGGCATCTATGCC
CRISPR2 colony 2    GCCGCGTGTGCGGAGACAGCAGCAGCAGGAAGC--TATGGCATCTATGCC
CRISPR2 colony 3    GCCGCGTGTGCGGAGACAGCAGCAGCGGGAAGCACTATGGCATCTATGCC
CRISPR2 colony 4    GCCGCGTGTGCGGAGACAGCAGCAGCAGGAAGC--TATGGCATCTATGCC
CRISPR2 colony 5    GCCGCGTGTGCGGAGACAGCAGCAGCGGGAAGCACTATGGCATCTATGCC
CRISPR2 colony 6    GCCGCGTGTGCGGAGACAGCAGCAGCAGGAAGC--TATGGCATCTATGCC
CRISPR2 colony 7    GCCGCGTGTGCGGAGACAGCAGCAGCGGGAAGCACTATGGCATCTATGCC
CRISPR2 colony 8    GCCGCGTGTGCGGAGACAGCAGCAGCAGGAAGC--TATGGCATCTATGCC
CRISPR2 colony 9    GCCGCGTGTGCGGAGACAGCAGCAGCGGGAAGCACTATGGCATCTATGCC
CRISPR2 colony 10   GCCGCGTGTGCGGAGACAGCAGCAGCGGGAAGCACTATGGCATCTATGCC
```

```
hNR2E3 gene         GCCGCGTGTGCGGAGACAGCAGCAGCGGGAAGCA-CTATGGCATCTATGC
CRISPR3 colony 1    GCCGCGTGTGCGGAGACAGCAGCAGCGGGAAGCA-CTATGGCATCTATGC
CRISPR3 colony 2    GCCGCGTGTGCGGAGACAGCAGCAGCAGGAAGCAACTATGGCATCTATGC
CRISPR3 colony 3    GCCGCGTGTGCGGAGACAGCAGCAGCAGGAAGCAACTATGGCATCTATGC
CRISPR3 colony 4    GCCGCGTGTGCGGAGACAGCAGCAGCAGGAAGCAACTATGGCATCTATGC
CRISPR3 colony 5    GCCGCGTGTGCGGAGACAGCAGCAGCAGGAAGCAACTATGGCATCTATGC
CRISPR3 colony 6    GCCGCGTGTGCGGAGACAGCAGCAGCGGGAAGCA-CTATGGCATCTATGC
CRISPR3 colony 7    GCCGCGTGTGCGGAGACAGCAGCAGCGGGAAGCA-CTATGGCATCTATGC
CRISPR3 colony 8    GCCGCGTGTGCGGAGACAGCAGCAGCAGGAAGCAACTATGGCATCTATGC
CRISPR3 colony 9    GCCGCGTGTGCGGAGACAGCAGCAGCAGGAAGCAACTATGGCATCTATGC
CRISPR3 colony 10   GCCGCGTGTGCGGAGACAGCAGCAGCGGGAAGCA-CTATGGCATCTATGC
```

Figure S1. Sequence alignment of the sub-cloned G56R-CRISPR2/3 alleles from 10 bacterial colonies. The top line of each alignment (in bold) shows the reference human *NR2E3* sequence. The c.166G nucleotide is in green. The c.166G>A transition in the mutant alleles is shown in red. Positions of indels (CA deletion in G56R-CRISPR2, and A insertion in G56R-CRISPR3) are indicated by dashes.

ESRRB

```
ESRRB_WT          1 ATGTTTGCAGGCGCCGGGCTGGGAGGCACCCCATGCCGCAAGAGCTACGAGGACTGTGCC
ESRRB_G56R        1 ATGTTTGCAGGCGCCGGGCTGGGAGGCACCCCATGCCGCAAGAGCTACGAGGACTGTGCC
ESRRB_CRISPR2     1 ATGTTTGCAGGCGCCGGGCTGGGAGGCACCCCATGCCGCAAGAGCTACGAGGACTGTGCC
ESRRB_CRISPR3     1 ATGTTTGCAGGCGCCGGGCTGGGAGGCACCCCATGCCGCAAGAGCTACGAGGACTGTGCC
ESRRB_CRISPR4     1 ATGTTTGCAGGCGCCGGGCTGGGAGGCACCCCATGCCGCAAGAGCTACGAGGACTGTGCC

ESRRB_WT         61 AGCGGCATCATGGAGGACTCGGCCATCAAGTGCGAGTACATGCTCAACGCCATCCCCAAG
ESRRB_G56R       61 AGCGGCATCATGGAGGACTCGGCCATCAAGTGCGAGTACATGCTCAACGCCATCCCCAAG
ESRRB_CRISPR2    61 AGCGGCATCATGGAGGACTCGGCCATCAAGTGCGAGTACATGCTCAACGCCATCCCCAAG
ESRRB_CRISPR3    61 AGCGGCATCATGGAGGACTCGGCCATCAAGTGCGAGTACATGCTCAACGCCATCCCCAAG
ESRRB_CRISPR4    61 AGCGGCATCATGGAGGACTCGGCCATCAAGTGCGAGTACATGCTCAACGCCATCCCCAAG

ESRRB_WT        121 CGCCTGTGCCTCGTGTGCGGGGACATTGCCTCTGGCTACCACTACGGCGTGG
ESRRB_G56R      121 CGCCTGTGCCTCGTGTGCGGGGACATTGCCTCTGGCTACCACTACGGCGTGG
ESRRB_CRISPR2   121 CGCCTGTGCCTCGTGTGCGGGGACATTGCCTCTGGCTACCACTACGGCGTGG
ESRRB_CRISPR3   121 CGCCTGTGCCTCGTGTGCGGGGACATTGCCTCTGGCTACCACTACGGCGTGG
ESRRB_CRISPR4   121 CGCCTGTGCCTCGTGTGCGGGGACATTGCCTCTGGCTACCACTACGGCGTGG
```

GPR135

```
GPR135_WT         1 GGAAAGCGTCCACATTCCTAGTCCGGTAGCCCTCCTCGCGGTTGCGCCCTAGGAGCATCG
GPR135_G56R       1 GGAAAGCGTCCACATTCCTAGTCCGGTAGCCCTCCTCGCGGTTGCGCCCTAGGAGCATCG
GPR135_CRISPR2    1 GGAAAGCGTCCACATTCCTAGTCCGGTAGCCCTCCTCGCGGTTGCGCCCTAGGAGCATCG
GPR135_CRISPR3    1 GGAAAGCGTCCACATTCCTAGTCCGGTAGCCCTCCTCGCGGTTGCGCCCTAGGAGCATCG
GPR135_CRISPR4    1 GGAAAGCGTCCACATTCCTAGTCCGGTAGCCCTCCTCGCGGTTGCGCCCTAGGAGCATCG

GPR135_WT        61 AAATGTTGGGATTGCGGATGGCGTAGATGACAGGGTTGATGGCCCCATTGGCCCAGGTCA
GPR135_G56R      61 AAATGTTGGGATTGCGGATGGCGTAGATGACAGGGTTGATGGCCCCATTGGCCCAGGTCA
GPR135_CRISPR2   61 AAATGTTGGGATTGCGGATGGCGTAGATGACAGGGTTGATGGCCCCATTGGCCCAGGTCA
GPR135_CRISPR3   61 AAATGTTGGGATTGCGGATGGCGTAGATGACAGGGTTGATGGCCCCATTGGCCCAGGTCA
GPR135_CRISPR4   61 AAATGTTGGGATTGCGGATGGCGTAGATGACAGGGTTGATGGCCCCATTGGCCCAGGTCA
```

```
GPR135_WT       121 GCCAGACGGCCACCACGCTGAGGAGCGAGGGGGCCTGCATGGTCTGGGCCTGCCGGGCGG
GPR135_G56R     121 GCCAGACGGCCACCACGCTGAGGAGCGAGGGGGCCTGCATGGTCTGGGCCTGCCGGGCGG
GPR135_CRISPR2  121 GCCAGACGGCCACCACGCTGAGGAGCGAGGGGGCCTGCATGGTCTGGGCCTGCCGGGCGG
GPR135_CRISPR3  121 GCCAGACGGCCACCACGCTGAGGAGCGAGGGGGCCTGCATGGTCTGGGCCTGCCGGGCGG
GPR135_CRISPR4  121 GCCAGACGGCCACCACGCTGAGGAGCGAGGGGGCCTGCATGGTCTGGGCCTGCCGGGCGG

GPR135_WT       181 CGGCCAGCAGCACCAGGAAGCAGTAGGGCCCCCAGCAGCAGATGACGAAGACGATCATGA
GPR135_G56R     181 CGGCCAGCAGCACCAGGAAGCAGTAGGGCCCCCAGCAGCAGATGACGAAGACGATCATGA
GPR135_CRISPR2  181 CGGCCAGCAGCACCAGGAAGCAGTAGGGCCCCCAGCAGCAGATGACGAAGACGATCATGA
GPR135_CRISPR3  181 CGGCCAGCAGCACCAGGAAGCAGTAGGGCCCCCAGCAGCAGATGACGAAGACGATCATGA
GPR135_CRISPR4  181 CGGCCAGCAGCACCAGGAAGCAGTAGGGCCCCCAGCAGCAGATGACGAAGACGATCATGA

GPR135_WT       241 TGAGGACGGTG
GPR135_G56R     241 TGAGGACGGTG
GPR135_CRISPR2  241 TGAGGACGGTG
GPR135_CRISPR3  241 TGAGGACGGTG
GPR135_CRISPR4  241 TGAGGACGGTG
```

SOX17

```
SOX17_WT          1 GCGCTGGGCCCCGAGGGCGGCCGCGTGGCCATGGACGGCCTGGGCCTCCAGTTCCCCGAG
SOX17_G56R        1 GCGCTGGGCCCCGAGGGCGGCCGCGTGGCCATGGACGGCCTGGGCCTCCAGTTCCCCGAG
SOX17_CRISPR2     1 GCGCTGGGCCCCGAGGGCGGCCGCGTGGCCATGGACGGCCTGGGCCTCCAGTTCCCCGAG
SOX17_CRISPR3     1 GCGCTGGGCCCCGAGGGCGGCCGCGTGGCCATGGACGGCCTGGGCCTCCAGTTCCCCGAG
SOX17_CRISPR4     1 GCGCTGGGCCCCGAGGGCGGCCGCGTGGCCATGGACGGCCTGGGCCTCCAGTTCCCCGAG

SOX17_WT         61 CAGGGCTTCCCCGCCGGCCCGCCGCTGCTGCCTCCGCACATGGGCGGCCACTACCGCGAC
SOX17_G56R       61 CAGGGCTTCCCCGCCGGCCCGCCGCTGCTGCCTCCGCACATGGGCGGCCACTACCGCGAC
SOX17_CRISPR2    61 CAGGGCTTCCCCGCCGGCCCGCCGCTGCTGCCTCCGCACATGGGCGGCCACTACCGCGAC
SOX17_CRISPR3    61 CAGGGCTTCCCCGCCGGCCCGCCGCTGCTGCCTCCGCACATGGGCGGCCACTACCGCGAC
SOX17_CRISPR4    61 CAGGGCTTCCCCGCCGGCCCGCCGCTGCTGCCTCCGCACATGGGCGGCCACTACCGCGAC

SOX17_WT        121 TGCCAGAG
SOX17_G56R      121 TGCCAGAG
SOX17_CRISPR2   121 TGCCAGAG
```

```
SOX17_CRISPR3  121  TGCCAGAG
SOX17_CRISPR4  121  TGCCAGAG
```

RP1-37C10.3

```
RP1_WT            1  GGTGCAGAACCCCCAGCCTGGGTGATGTCACGGCCCCTTCAGTGCTGCCTTTCTGGGATC
RP1_G56R          1  GGTGCAGAACCCCCAGCCTGGGTGATGTCACGGCCCCTTCAGTGCTGCCTTTCTGGGATC
RP1_CRISPR2       1  GGTGCAGAACCCCCAGCCTGGGTGATGTCACGGCCCCTTCAGTGCTGCCTTTCTGGGATC
RP1_CRISPR3       1  GGTGCAGAACCCCCAGCCTGGGTGATGTCACGGCCCCTTCAGTGCTGCCTTTCTGGGATC
RP1_CRISPR4       1  GGTGCAGAACCCCCAGCCTGGGTGATGTCACGGCCCCTTCAGTGCTGCCTTTCTGGGATC

RP1_WT           61  CAGAGGAGCCCCCTCAGGCTTCACTCTCGTGCTTCCTGCTGCTGCAAATAGCCCGGGTCT
RP1_G56R         61  CAGAGGAGCCCCCTCAGGCTTCACTCTCGTGCTTCCTGCTGCTGCAAATAGCCCGGGTCT
RP1_CRISPR2      61  CAGAGGAGCCCCCTCAGGCTTCACTCTCGTGCTTCCTGCTGCTGCAAATAGCCCGGGTCT
RP1_CRISPR3      61  CAGAGGAGCCCCCTCAGGCTTCACTCTCGTGCTTCCTGCTGCTGCAAATAGCCCGGGTCT
RP1_CRISPR4      61  CAGAGGAGCCCCCTCAGGCTTCACTCTCGTGCTTCCTGCTGCTGCAAATAGCCCGGGTCT

RP1_WT          121  CGTGAGCCTGTGATGCAC
RP1_G56R        121  CGTGAGCCTGTGATGCAC
RP1_CRISPR2     121  CGTGAGCCTGTGATGCAC
RP1_CRISPR3     121  CGTGAGCCTGTGATGCAC
RP1_CRISPR4     121  CGTGAGCCTGTGATGCAC
```

NPEPL1/STX16-NPEPL1

```
NPELP1_WT        1  GGGTTGCTCCGGCCGGGCCTCTCCACCCTGCCCCATCGCCTGTCACTATTGCTGTCTGCA
NPELP1_G56R      1  GGGTTGCTCCGGCCGGGCCTCTCCACCCTGCCCCATCGCCTGTCACTATTGCTGTCTGCA
NPELP1_CRISPR2   1  GGGTTGCTCCGGCCGGGCCTCTCCACCCTGCCCCATCGCCTGTCACTATTGCTGTCTGCA
NPELP1_CRISPR3   1  GGGTTGCTCCGGCCGGGCCTCTCCACCCTGCCCCATCGCCTGTCACTATTGCTGTCTGCA
NPELP1_CRISPR4   1  GGGTTGCTCCGGCCGGGCCTCTCCACCCTGCCCCATCGCCTGTCACTATTGCTGTCTGCA

NPELP1_WT       61  AGGAGCTCGGGGTGGCTGGGAGGGAGCGTGTGGGGAGGCAGCAGAAGCTGGGCTGGGAGG
NPELP1_G56R     61  AGGAGCTCGGGGTGGCTGGGAGGGAGCGTGTGGGGAGGCAGCAGAAGCTGGGCTGGGAGG
NPELP1_CRISPR2  61  AGGAGCTCGGGGTGGCTGGGAGGGAGCGTGTGGGGAGGCAGCAGAAGCTGGGCTGGGAGG
```

```
NPELP1_CRISPR3 61  AGGAGCTCGGGGTGGCTGGGAGGGAGCGTGTGGGGAGGCAGCAGAAGCTGGGCTGGGAGG
NPELP1_CRISPR4 61  AGGAGCTCGGGGTGGCTGGGAGGGAGCGTGTGGGGAGGCAGCAGAAGCTGGGCTGGGAGG
```

TSPAN4

```
TSPAN4_WT         1 ACCTTGTCCGTGTAGGCGAAGAAGAGGATGGCGATGGTGGCCTCCAGCAGGAACACCAGC
TSPAN4_G56R       1 ACCTTGTCCGTGTAGGCGAAGAAGAGGATGGCGATGGTGGCCTCCAGCAGGAACACCAGC
TSPAN4_CRISPR2    1 ACCTTGTCCGTGTAGGCGAAGAAGAGGATGGCGATGGTGGCCTCCAGCAGGAACACCAGC
TSPAN4_CRISPR3    1 ACCTTGTCCGTGTAGGCGAAGAAGAGGATGGCGATGGTGGCCTCCAGCAGGAACACCAGC
TSPAN4_CRISPR4    1 ACCTTGTCCGTGTAGGCGAAGAAGAGGATGGCGATGGTGGCCTCCAGCAGGAACACCAGC

TSPAN4_WT        61 AGCAGCAGCAGGAAGAACTGTGGAGGGGGCAGGCTCAGACAGGGACCCGAAAGGCCGCAC
TSPAN4_G56R      61 AGCAGCAGCAGGAAGAACTGTGGAGGGGGCAGGCTCAGACAGGGACCCGAAAGGCCGCAC
TSPAN4_CRISPR2   61 AGCAGCAGCAGGAAGAACTGTGGAGGGGGCAGGCTCAGACAGGGACCCGAAAGGCCGCAC
TSPAN4_CRISPR3   61 AGCAGCAGCAGGAAGAACTGTGGAGGGGGCAGGCTCAGACAGGGACCCGAAAGGCCGCAC
TSPAN4_CRISPR4   61 AGCAGCAGCAGGAAGAACTGTGGAGGGGGCAGGCTCAGACAGGGACCCGAAAGGCCGCAC

TSPAN4_WT       121 AGCCTCCGCCCCACAGCATGCCAGGGGCCA
TSPAN4_G56R     121 AGCCTCCGCCCCACAGCATGCCAGGGGCCA
TSPAN4_CRISPR2  121 AGCCTCCGCCCCACAGCATGCCAGGGGCCA
TSPAN4_CRISPR3  121 AGCCTCCGCCCCACAGCATGCCAGGGGCCA
TSPAN4_CRISPR4  121 AGCCTCCGCCCCACAGCATGCCAGGGGCCA
```

SLC39A4

```
SLC39A4_WT        1 TAGGTTTGTGAGGTGTGGGATCTTAAGTCAAAGGTGGGGGACTAGGGCAGGGTATCAGAA
SLC39A4_G56R      1 TAGGTTTGTGAGGTGTGGGATCTTAAGTCAAAGGTGGGGGACTAGGGCAGGGTATCAGAA
SLC39A4_CRISPR2   1 TAGGTTTGTGAGGTGTGGGATCTTAAGTCAAAGGTGGGGGACTAGGGCAGGGTATCAGAA
SLC39A4_CRISPR3   1 TAGGTTTGTGAGGTGTGGGATCTTAAGTCAAAGGTGGGGGACTAGGGCAGGGTATCAGAA
SLC39A4_CRISPR4   1 TAGGTTTGTGAGGTGTGGGATCTTAAGTCAAAGGTGGGGGACTAGGGCAGGGTATCAGAA

SLC39A4_WT       61 GGTGATGTCATCCTCGTACAGGGACAGCAGCAGCAGGACGGTCCAGCCGCCCAGCAGGCC
SLC39A4_G56R     61 GGTGATGTCATCCTCGTACAGGGACAGCAGCAGCAGGACGGTCCAGCCGCCCAGCAGGCC
SLC39A4_CRISPR2  61 GGTGATGTCATCCTCGTACAGGGACAGCAGCAGCAGGACGGTCCAGCCGCCCAGCAGGCC
```

```
SLC39A4_CRISPR3 61  GGTGATGTCATCCTCGTACAGGGACAGCAGCAGCAGGACGGTCCAGCCGCCCAGCAGGCC
SLC39A4_CRISPR4 61  GGTGATGTCATCCTCGTACAGGGACAGCAGCAGCAGGACGGTCCAGCCGCCCAGCAGGCC
```

TBX5-AS1

```
TBX5_WT           1  CAGTCCAGGCAACTTTTTTCTTTGCCTGGGGCCATTTCTTCTAAATGGAAGGAGAAAAGT
TBX5_G56R         1  CAGTCCAGGCAACTTTTTTCTTTGCCTGGGGCCATTTCTTCTAAATGGAAGGAGAAAAGT
TBX5_CRISPR2      1  CAGTCCAGGCAACTTTTTTCTTTGCCTGGGGCCATTTCTTCTAAATGGAAGGAGAAAAGT
TBX5_CRISPR3      1  CAGTCCAGGCAACTTTTTTCTTTGCCTGGGGCCATTTCTTCTAAATGGAAGGAGAAAAGT
TBX5_CRISPR4      1  CAGTCCAGGCAACTTTTTTCTTTGCCTGGGGCCATTTCTTCTAAATGGAAGGAGAAAAGT

TBX5_WT          61  CTGAGCATTAGAAATTGTGGCATTAATGTCTGCAAGGGCTAATGGAGTGAAAAGAATACA
TBX5_G56R        61  CTGAGCATTAGAAATTGTGGCATTAATGTCTGCAAGGGCTAATGGAGTGAAAAGAATACA
TBX5_CRISPR2     61  CTGAGCATTAGAAATTGTGGCATTAATGTCTGCAAGGGCTAATGGAGTGAAAAGAATACA
TBX5_CRISPR3     61  CTGAGCATTAGAAATTGTGGCATTAATGTCTGCAAGGGCTAATGGAGTGAAAAGAATACA
TBX5_CRISPR4     61  CTGAGCATTAGAAATTGTGGCATTAATGTCTGCAAGGGCTAATGGAGTGAAAAGAATACA

TBX5_WT         121  TTATTCTCCTTTTCTTCTTAGAAAAAAATTT
TBX5_G56R       121  TTATTCTCCTTTTCTTCTTAGAAAAAAATTT
TBX5_CRISPR2    121  TTATTCTCCTTTTCTTCTTAGAAAAAAATTT
TBX5_CRISPR3    121  TTATTCTCCTTTTCTTCTTAGAAAAAAATTT
TBX5_CRISPR4    121  TTATTCTCCTTTTCTTCTTAGAAAAAAATTT
```

FAM69B/SNHG7

```
FAM69B_WT         1  TGGCCCGCCAAGGCCACGCAGGCCCCAGCCACTGCTGGAGAGCACCCTGCAGGGCAGGCG
FAM69B_G56R       1  TGGCCCGCCAAGGCCACGCAGGCCCCAGCCACTGCTGGAGAGCACCCTGCAGGGCAGGCG
FAM69B_CRISPR2    1  TGGCCCGCCAAGGCCACGCAGGCCCCAGCCACTGCTGGAGAGCACCCTGCAGGGCAGGCG
FAM69B_CRISPR3    1  TGGCCCGCCAAGGCCACGCAGGCCCCAGCCACTGCTGGAGAGCACCCTGCAGGGCAGGCG
FAM69B_CRISPR4    1  TGGCCCGCCAAGGCCACGCAGGCCCCAGCCACTGCTGGAGAGCACCCTGCAGGGCAGGCG
FAM69B_Ref        1  TGGCCCGCCAAGGCCACGCAGGCCCCAGCCACTGCTGGAGAGCACCCTGCAGGGCAGGCG

FAM69B_WT        61  GCAGCAGTGGGCGCAACAGGGGTGGGAGGGCGGCCGCGTGCCAGGCGCCATGCGGCACGC
FAM69B_G56       61  GCAGCAGTGGGCGCAACAGGGGTGGAAGGGCGGCCGCGTGCCAGGCGCCATGCGGCACGC
```

```
FAM69B_CRISPR2  61  GCAGCAGTGGGCGCAACAGGGGTGGAAGGGCGGCCGCGTGCCAGGCGCCATGCGGCACGC
FAM69B_CRISPR3  61  GCAGCAGTGGGCGCAACAGGGGTGGAAGGGCGGCCGCGTGCCAGGCGCCATGCGGCACGC
FAM69B_CRISPR4  61  GCAGCAGTGGGCGCAACAGGGGTGGAAGGGCGGCCGCGTGCCAGGCGCCATGCGGCACGC
FAM69B_Ref      61  GCAGCAGTGGGCGCAACAGGGGTGGAAGGGCGGCCGCGTGCCAGGCGCCATGCGGCACGC

FAM69B_WT      121  CTTCGGTGAGGTAGAGGTCCCCACAGTAGCCCAGCAGTCTGGAGGCGTGCTCCTTCTCCT
FAM69B_G56R    121  CCTCGGTGAGGTAGAGGTCCCCACAGTAGCCCAGCAGTCTGGAGGCGTGCTCCTTCTCCT
FAM69B_CRISPR2 121  CCTCGGTGAGGTAGAGGTCCCCACAGTAGCCCAGCAGTCTGGAGGCGTGCTCCTTCTCCT
FAM69B_CRISPR3 121  CCTCGGTGAGGTAGAGGTCCCCACAGTAGCCCAGCAGTCTGGAGGCGTGCTCCTTCTCCT
FAM69B_CRISPR4 121  CCTCGGTGAGGTAGAGGTCCCCACAGTAGCCCAGCAGTCTGGAGGCGTGCTCCTTCTCCT
FAM69B_Ref     121  CCTCGGTGAGGTAGAGGTCCCCACAGTAGCCCAGCAGTCTGGAGGCGTGCTCCTTCTCCT

FAM69B_WT      181  GCAGGGACAGCAGCAGCAGGAACTCGTTACGCTGCAGCAGGGCCCACACGGACTTGGCTT
FAM69B_G56R    181  GCAGGGACAGCAGCAGCAGGAACTCGTTACGCTGCAGCAGGGCCCACACGGACTTGGCTT
FAM69B_CRISPR2 181  GCAGGGACAGCAGCAGCAGGAACTCGTTACGCTGCAGCAGGGCCCACACGGACTTGGCTT
FAM69B_CRISPR3 181  GCAGGGACAGCAGCAGCAGGAACTCGTTACGCTGCAGCAGGGCCCACACGGACTTGGCTT
FAM69B_CRISPR4 181  GCAGGGACAGCAGCAGCAGGAACTCGTTACGCTGCAGCAGGGCCCACACGGACTTGGCTT
FAM69B_Ref     181  GCAGGGACAGCAGCAGCAGGAACTCGTTACGCTGCAGCAGGGCCCACACGGACTTGGCTT

FAM69B_WT      241  CCGCCAGGGAC
FAM69B_G56R    241  CCGCCAGGGAC
FAM69B_CRISPR2 241  CCGCCAGGGAC
FAM69B_CRISPR3 241  CCGCCAGGGAC
FAM69B_CRISPR4 241  CCGCCAGGGAC
FAM69B_Ref     241  CCGCCAGGGAC
```

PDLIM7

```
PDLIM7_WT        1  AGGTGGGCGGCCAGGGCCAGGCCAGCAGGAGGCTCAGGCCAGGGGCACGAAAGGGGGGGT
PDLIM7_G56R      1  AGGTGGGCGGCCAGGGCCAGGCCAGCAGGAGGCTCAGGCCAGGGGCACGAAAGGGGGGGT
PDLIM7_CRISPR2   1  AGGTGGGCGGCCAGGGCCAGGCCAGCAGGAGGCTCAGGCCAGGGGCACGAAAGGGGGGGT
PDLIM7_CRISPR3   1  AGGTGGGCGGCCAGGGCCAGGCCAGCAGGAGGCTCAGGCCAGGGGCACGAAAGGGGGGGT
PDLIM7_CRISPR4   1  AGGTGGGCGGCCAGGGCCAGGCCAGCAGGAGGCTCAGGCCAGGGGCACGAAAGGGGGGGT
```

```
PDLIM7_WT        61 GAGGTAGGCAGAAGCTGGAGGCACTAAGGGGGTGCCCTGGGCAGGAGGAACAGAAAGAGG
PDLIM7_G56R      61 GAGGTAGGCAGAAGCTGGAGGCACTAAGGGGGTGCCCTGGGCAGGAGGAACAGAAAGAGG
PDLIM7_CRISPR2   61 GAGGTAGGCAGAAGCTGGAGGCACTAAGGGGGTGCCCTGGGCAGGAGGAACAGAAAGAGG
PDLIM7_CRISPR3   61 GAGGTAGGCAGAAGCTGGAGGCACTAAGGGGGTGCCCTGGGCAGGAGGAACAGAAAGAGG
PDLIM7_CRISPR4   61 GAGGTAGGCAGAAGCTGGAGGCACTAAGGGGGTGCCCTGGGCAGGAGGAACAGAAAGAGG

PDLIM7_WT       121 GAGGCAGAGAGGGCAGCCGGCTGGAGAGGGC
PDLIM7_G56R     121 GAGGCAGAGAGGGCAGCCGGCTGGAGAGGGC
PDLIM7_CRISPR2  121 GAGGCAGAGAGGGCAGCCGGCTGGAGAGGGC
PDLIM7_CRISPR3  121 GAGGCAGAGAGGGCAGCCGGCTGGAGAGGGC
PDLIM7_CRISPR4  121 GAGGCAGAGAGGGCAGCCGGCTGGAGAGGGC
```

Figure S2. Sequencing analysis of the predicted off-targets regions for gRNA1. The top 10 off-target exons predicted by the CRISPOR software were sequenced in the G56R-CRISPR2/3/4 iPSC lines and showed sequences identical to a WT iPSC line. Two silent nucleotide changes in *FAM69B* were detected in the WT clone (indicated in white) as compared to the reference sequence.
